# On‐Chip Monolithically Integrated Ultraviolet Low‐Threshold Plasmonic Metal‒Semiconductor Heterojunction Nanolasers

**DOI:** 10.1002/advs.202301493

**Published:** 2023-08-09

**Authors:** Jia‐Yuan Sun, Duc Huy Nguyen, Jia‐Ming Liu, Chia‐Yao Lo, Yuan‐Ron Ma, Yi‐Jia Chen, Jui‐Yun Yi, Jian‐Zhi Huang, Hien Giap, Hai Yen Thi Nguyen, Chun‐Da Liao, Ming‐Yi Lin, Chien‐Chih Lai

**Affiliations:** ^1^ Department of Physics National Dong Hwa University Hualien 974301 Taiwan; ^2^ Department of Electrical and Computer Engineering University of California Los Angeles CA 90095 USA; ^3^ Institute of Photonics National Yang Ming Chiao Tung University Tainan 711010 Taiwan; ^4^ Institute of Optoelectronics National Chung Hsing University Taichung 402202 Taiwan; ^5^ Department of Optoelectronics and Materials Technology National Taiwan Ocean University Keelung 202301 Taiwan; ^6^ Department of Materials Science and Engineering National Dong Hwa University Hualien 974301 Taiwan; ^7^ Department of Electrical Engineering National Kaohsiung Normal University Kaohsiung 824004 Taiwan; ^8^ Department of Opto‐Electronic Engineering National Dong Hwa University Hualien 974301 Taiwan; ^9^ R&D Center Taiwan Semiconductor Manufacturing Company Hsinchu 300091 Taiwan; ^10^ Department of Dermatology National Taiwan University Hospital and College of Medicine National Taiwan University Taipei 100229 Taiwan

**Keywords:** heterojunction, nanolaser, nanowire, plasmonics, ultraviolet

## Abstract

The metal‒semiconductor heterojunction is imperative for the realization of electrically driven nanolasers for chip‐level platforms. Progress in developing such nanolasers has hitherto rarely been realized, however, because of their complexity in heterojunction fabrication and the need to use noble metals that are incompatible with microelectronic manufacturing. Most plasmonic nanolasers lase either above a high threshold (10^1^‒10^3^ MW cm^−2^) or at a cryogenic temperature, and lasing is possible only after they are removed from the substrate to avoid the large ohmic loss and the low modal reflectivity, making monolithic fabrication impossible. Here, for the first time, record‐low‐threshold, room‐temperature ultraviolet (UV) lasing of plasmon‐coupled core‒shell nanowires that are directly grown on silicon is demonstrated. The naturally formed core‒shell metal‒semiconductor heterostructure of the nanowires leads to a 100‐fold improvement in growth density over previous results. This unprecedentedly high nanowire density creates intense plasmonic resonance, which is outcoupled to the resonant Fabry‒Pérot microcavity. By boosting the emission strength by a factor of 100, the hybrid photonic‒plasmonic system successfully facilitates a record‐low laser threshold of 12 kW cm^−2^ with a spontaneous emission coupling factor as high as ≈0.32 in the 340‒360 nm range. Such architecture is simple and cost‐competitive for future UV sources in silicon integration.

## Graphical abstract

By boosting the emission strength by a factor of 100, a new hybrid photonic‒plasmonic architecture is successfully demonstrated to realize low‐threshold room‐temperature UV nanolasers that can be monolithically integrated into silicon. The synergistic engineering of the metal‒semiconductor heterojunction, the modal reflectivity, and the intermodal coupling engender the realization of electrically driven nanolasers.

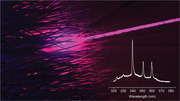

## 1 Introduction

With the booming interest in nanotechnology and nanoscience, strong demands for tight interconnection between on‐chip photonics and electronics have spurred pronounced studies into the development of nanolasers. The limited progress in ultraviolet (UV) nanolasers so far is caused by challenges in extending the emission to short wavelengths due to the high optical absorption of existing nanolaser materials in the UV region, particularly at wavelengths below 360 nm (near the bandgap of gallium nitride (GaN)). To date, lasing of nanolasers in the UV region is a long‐standing goal that has been eagerly pursued in white light, information storage, and biomedical science across a wide variety of applications.^[^ ^1^, ^2^, ^3^ ^]^ In light of this, the exploration of materials emitting in the UV spectral region as relevant alternatives to the existing GaN and aluminium GaN (AlGaN) materials is essential to achieving UV nanolasers, that are compatible with the fabrication requirement of silicon‐based on‐chip microelectronic manufacturing. For this purpose, two recognized requirements have to be fulfilled: i) An easily accessible wide‐bandgap optical gain medium for monolithic integration with Si has to be developed. ii) Efficient UV lasers operating at room temperature (RT) require effective cavity feedback, efficient optical amplification, and high optical gain. These two requirements are of concern to ultracompact all‐optical interconnect on silicon (Si).^[^ ^4^ ^]^

In place of using bulky solid‐state lasers via off‐chip nonlinear frequency conversions for UV laser emission, the use of wide‐bandgap materials as active nano emitters that emit directly in the UV region engenders superior features, including miniaturization and compatibility with chip‐level integration. Within the family of wide‐bandgap materials, zinc oxide (ZnO) is one of the most widely used metallic oxide semiconductors for UV light‐emitting diodes and laser diodes because of its naturally wide bandgap in the near‐UV region and its large exciton binding energy. However, the poor crystal quality and defect‐related deep‐level emission of the p‐type wide‐bandgap ZnO are of great concern,^[^ ^5^ ^]^ which precludes the interest in developing UV‐luminescent homojunction devices through a facile route at a competitive cost (Table S1). As an alternative, group III‒nitride semiconductors have proven feasible under optical pumping in the UV band (Table S2). However, some problems with the Al‐rich AlGaN epilayers arise with regard to their low crystal quality, high dislocation density, and large residual strain. Moreover, owing to the delicate growth procedure and the prerequisite expensive equipment, successful development of GaN nanolasers that lase at wavelengths shorter than 360 nm has hitherto been hindered, thus severely restricting the feasibility of GaN nanolasers for practical applications (Table S3).

Among the existing nanolaser schemes, metal‒semiconductor heterojunctions and plasmonics are at the heart of present electrically driven nano‐electronics and nano‐photonics. Despite the success in realizing nanolasers using either metal‒insulator‒metal or metal‒insulator‒semiconductor architectures,^[^ ^6^, ^7^ ^]^ the insulating barrier limits the implementation of on‐chip electrically driven lasers. Moreover, the abovementioned success significantly depends on ultra‐flat single‐crystalline metal films that require atomically controlled growth involving expensive interfacial epitaxy.^[^ ^8^, ^9^, ^10^, ^11^, ^12^ ^]^ This severely hinders the development of facile and cost‐competitive fabrication processes for future mass production. The ideal feature of a plasmon‐enhanced UV laser with a metal‒semiconductor heterojunction scheme is a spectral overlap between the surface plasmon resonance (SPR) of the hot spot and the photoluminescence (PL) of the gain medium. Thus, the combination of bandgap engineering and surface‐plasmon‐enhanced luminescence in heterostructures has been considered as an effective bridge between on‐chip nano‐photonics and micro‐photonics. However, this spectral overlap requires bandgap alignment and shifts, which are difficult to accomplish. For most plasmonic nanolasers that operate after being separated from the substrate, either pumping above a megawatt cm^−2^‐level threshold or operating at a cryogenic temperature is needed to overcome the large ohmic losses and the low modal reflectivity. These limitations make monolithic fabrication impossible. To this end, the design of effective cavity feedback of SPR and the use of accessible laser materials that are compatible with existing Si‐based photonics are highly desirable.

Herein, we focus on the use of the low‐cost and semiconductor‐manufacturing‐compatible bismuth (Bi) as a UV hybrid photonic‒plasmonic host.^[^ ^13^, ^14^, ^15^, ^16^, ^17^ ^]^ To date, it remains challenging to fabricate large quantities of high‐quality 1D Bi nanostructures.^[^ ^18^, ^19^, ^20^ ^]^ In this work, we experimentally demonstrate a fabrication process of combining the UV SPR‐coupled architecture with eco‐friendly and low‐cost Bi nanowires (NWs) by using a simple, scalable, and template‐free vapor‒solid (VS) technique.^[^ ^21^, ^22^, ^23^, ^24^, ^25^ ^]^ The developed and modified VS technique requires neither an inert environment, nor any costly noble metal, nor an epitaxial system. It thus possesses many attractive advantages such as a low cost and a fast growth rate. By exposing the Bi NWs to an oxygen‐containing atmosphere, a self‐terminating bismuth oxide (Bi_2_O_3_) shell naturally forms on each of the as‐grown high‐yield Bi NWs due to its negative Gibbs free energy.^[^ ^26^, ^27^, ^28^ ^]^ As the gain medium, the NWs have the core‒shell metal‒semiconductor heterostructure with a 100‐fold improvement in density over existing gain materials for UV emission.

Specifically, the UV SPR excited at the Bi/Bi_2_O_3_ heterojunction interface can modulate the local field and further overcome the optical loss. It is then outcoupled to the Fabry‒Pérot (F‒P) lasing modes. Hence, the Bi NW lasers outperform other NW lasers by simultaneously providing a low lasing threshold of ≈12 kW cm^−2^ and a remarkably high spontaneous emission coupling factor *β* of ≈0.32 at RT. In contrast to most single‐NW lasers, the enhanced Fresnel reflection of ensemble NWs can suppress the optical loss and preferentially enhance the radiative photons by up to 100 folds through a hybrid SPR‐coupled F‒P microcavity, suitable for monolithical on‐chip silicon integration. It is in principle an ideal form for the nanolasers in light of the essential requirements of facile fabrications, competitive cost, and effective miniaturization for industrial mass production.

## 2 Results and Discussion

### 2.1 Investigation of Core‒Shell Metal‒Semiconductor Heterojunction NWs

Numerous modified growth techniques have been developed over the past decades; however, each of these approaches suffered from expensive equipment, time‐consuming procedures, or complexity from vapor deposition. Here, a controlled low‐cost VS technique, by which target sources are deposited on a crystalline substrate, is the key to delivering dense Bi‐core Bi_2_O_3_‐shell heterojunction NWs on a Si substrate. Thermodynamically, the high‐throughput VS growth in our work can be ascribed to a synergistic effect from the small millimeter distance and the large thermally‐induced stress at a high temperature (Figure S1, in which the thermally induced compressive strain occurs between the Bi film and the substrate). The details of the fabrication, characterization, and growth of these NWs are described in Figures S2–S6, Sections S1–S3, and Section S8 (Supporting Information).

**Figure 1:**
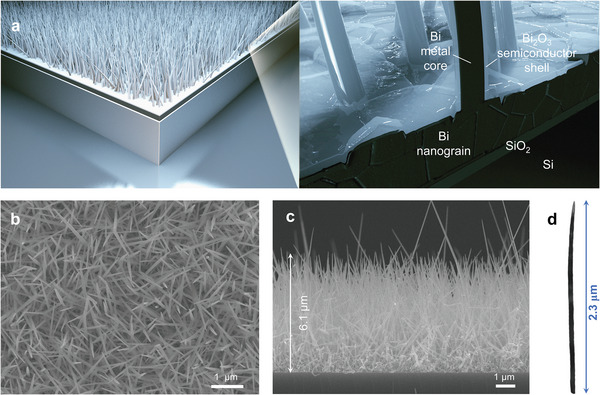
Morphological characterizations of a core‒shell metal‒semiconductor heterojunction NW device. a) Schematic of a dense and oriented NW array realized by the scalable, high‐yield, and template‐free VS technique. b,c) Planar‐ and side‐view SEM images of densely packed heterojunction NWs directly grown on silicon, showing an average thickness of ≈6.1 µm. d) Low‐magnification TEM image of a single truncated NW with a conical tapering as further resolved in Figure 2.

**Figure 2:**
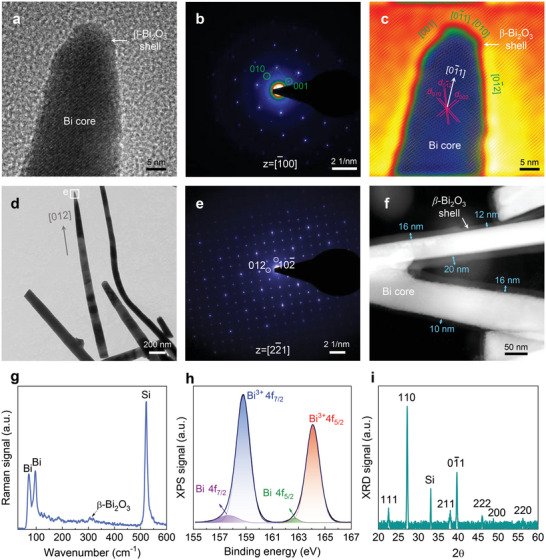
Nanostructure characterizations of the core‒shell metal‒semiconductor heterojunction NWs fabricated by the high‐yield VS technique and the high‐temperature treatment. a) Atomic lattice image coupled with b) its SAED pattern of the NW in Figure 1d, indicating that the Bi crystal core is free of defect and encapsulated by an amorphous β‐Bi_2_O_3_ thin shell with a tapered feature. c) Reconstructed lattice image of (a) by the inverse Fourier transform using all the diffraction spots revealing a high crystallinity with a rhombohedral structure having well‐developed crystalline facets in the [$\bar{1}$00] zone axis. d,e) Low‐magnification TEM image showing a growth direction along the [012] of the hexagonal structure as outlined by its SAED pattern in (e). f) Representative high‐angle annular dark‐field image showing a bifurcated core surrounded by a protective shell with an amorphous signature. g) Raman signal showing a predominant crystalline Bi core with an amorphous β‐Bi_2_O_3_ shell having a frequency‐shift than those of Bi bulk (refer to text). h) XPS spectrum of the same sample in Figure 1. Note that no relevant signals of bi‐, tetra‐, or penta‐valent states were detectable in all the samples, implying that the oxide shell consists primarily of a Bi (III) oxidation state. i) XRD trace with sharp diffraction of the orientations labeled, indicating the crystallization of metallic Bi (space group: R$\bar{3}$m) of rhombohedral structure on a cubic Si substrate.

A device configuration, consisting primarily of a thin native SiO_2_ layer along with Bi nanograins beneath dense Bi‐core Bi_2_O_3_‐shell heterojunction NWs, is schematically illustrated in **Figure** **1**a. For the sample analyzed here, the average thickness of the core‒shell heterojunction NWs is ≈6.1 µm and the average diameter is ≈50‒100 nm, corresponding to a surprisingly high density of ≈3×10^9^ cm^−2^ (refer to Discussion in Supporting Information for details), as assessed from planar‐view and side‐view scanning electron microscopy (SEM) images (Figure 1b,c; Figures S4 and S6, Supporting Information) together with lengthwise cross‐sectional transmission electron microscopy (TEM) images (**Figure** **2** and Figure S5, Supporting Information). Since all the NWs are oblique, the average length of the NWs is greater than the thickness. By scrutinizing >20 cross‐sectional SEM images, the average length of the core‒shell heterojunction NWs is found to be ≈7.8 µm.

In the low magnification high‐resolution TEM (HRTEM) image shown in Figure 1d, the truncated core‒shell NW exhibits a strong tapering located near the apex, giving a conical growth front of a high aspect ratio (i.e., length‐to‐diameter ratio). The highest aspect ratio is 78 to 156 (i.e., 7.8 µm/100 nm to 7.8 µm/50 nm). As a promising alternative to costly indium tin oxide, high aspect‐ratio NWs as transparent conducting electrodes have demonstrated exceptional levels of electrical performance and mechanical flexibility.^[^ ^29^, ^30^, ^31^, ^32^ ^]^ However, the facile, low‐cost, and reproducible production of extremely dense and high aspect‐ratio NWs, which is critical for achieving novel nanodevices, remains challenging.

Our strategy presented here differs conceptually from previous studies on forming low‐density Bi NWs. The previously recorded values of Bi NW density were of the order of only 10^4^ to 10^7^ cm^−2^.^[^ ^20^, ^33^, ^34^ ^]^ Thanks to the millimeter‐spacing sublimation and the high‐temperature‐induced stress, it was expected that there would be a thin supporting Bi film containing a substantial amount of tiny grains (Figures S4 and S5, Supporting Information).^[^ ^35^ ^]^ The presence of tiny grains promotes a high growth density that is different from the low growth density resulting from larger grain sizes (i.e., thicker Bi film).^[^ ^36^ ^]^ Figure 2a shows the HRTEM image of a representative NW, and Figure 2b,c shows the corresponding selected area electron diffraction (SAED) pattern and restructured lattice image, respectively. These images indicate an atomically abrupt core‒shell metal‒semiconductor heterojunction. The core arranges in a close‐packed manner of rhombohedral Bi lattice as well as in the reciprocal [0$\bar{1}$1] growth direction as reported earlier.^[^ ^33^, ^35^, ^36^, ^37^, ^38^, ^39^, ^40^, ^41^ ^]^ Note that the [0$\bar{1}$1] direction in the rhombohedral structure is [110] in the hexagonal structure. Sharp and bright SAED spots are clearly visible in Figure 2b, demonstrating a high‐crystallinity Bi core that is predominantly perpendicular to the [$\bar{1}$00] zone axis when viewed edge‐on. The shell formed by native oxidation is a self‐terminating Bi_2_O_3_ semiconductor, which preserves the Bi crystalline core, as found for aluminium, copper, and gallium, respectively.^[^ ^42^, ^43^, ^44^ ^]^ This result is consistent with the Raman and X‐ray diffraction (XRD) data (addressed in the following).

Further examination of certain NW apexes using TEM shows another growth direction along <012> in the hexagonal scheme (Figure 2d,e, and Figure S5, Supporting Information), besides aforementioned predominant <110> for a Bi NW. This protective oxide shell of a thickness of a few to tens of nanometers also acts as the active luminescent gain region for this study (Figure 2f and Figure S5, Supporting Information), as discussed further in the following. It is also noteworthy that, on careful inspection using SEM (Figure S6, Supporting Information), the presence of cross‐over originates from the difference in anisotropic growth rates that account for the length differences among NWs shown in Figure 1c, by analogy to the NWs grown by the physical vapor deposition.^[^ ^45^ ^]^ These evidence also supports that the Bi NWs were grown through the preferential surface diffusion of Bi atoms from their base Bi thin film in such a manner that they have well‐developed crystalline facets at the core–shell interface. From the above atomic‐scale inspections, it can be concluded that the core‒shell NWs presented here have these prominent features: i) the core–shell interface is atomically sharp, and the shape and growth facet are well‐defined; ii) each core‒shell NW shows high crystallinity, thus superb plasmonic properties essential for an efficient nanophotonic device; iii) the proposed and demonstrated synthesis method is facile and easy to cost‐effectively scale up for the present Si manufacturing on account of its simple process and high‐yield nature without using sophisticated equipment.

Although HRTEM is a promising technique for the characterization of the degree of crystallinity of NWs, it is rather difficult to verify the overall crystalline quality on the micrometer scale by using this technique. Alternatively, the confocal Raman microscopy with its direct and rapid testing nature has been employed to study the NW quality with a submicron spatial resolution. Based on the Gaussian beam relation (refer to Section S5, Supporting Information), we can estimate the focus spot diameter at the 633‐nm wavelength to be 310 nm (or a radius of 155 nm), which contains a few NWs. In the experiment, a longer exposure time of several minutes was required to acquire substantial Raman signals. As presented in Figure 2g, two prominent Raman peaks can be specified based on the group theory prediction. The doubly degenerate *E*_g_ (≈72 cm^−1^) and the non‐degenerate *A*_1_ *_g_* active modes (≈97 cm^−1^) are due to the Bi metal core, whereas the broad and faint vibration hump (≈312 cm^−1^) belongs to featured Bi‐O bond stretches and can be assigned to the β‐Bi_2_O_3_ semiconductor shell,^[^ ^46^ ^]^ as identified by the HRTEM images mentioned above. The observed Bi Raman peaks exhibit a frequency shift as compared to those of Bi in the bulk form (71 and 98 cm^−1^);^[^ ^47^ ^]^ this frequency shift may be elucidated by quantum confinement effects in view of a relatively large length‐to‐diameter ratio,^[^ ^48^, ^49^, ^50^ ^]^ as described above.

To further confirm the presence of an oxide phase within the NWs, the quantitative compositions of the NWs were analyzed by X‐ray photoelectron spectroscopy (XPS) recorded over a millimeter‐sized area. Figure 2h shows the high‐resolution XPS spectrum of the Bi 4f state of an as‐grown core‒shell metal‒semiconductor heterojunction NW sample fitted with a Gaussian shape, which reveals two symmetrical oxide shell spectra (Bi_2_O_3_ 4f_7/2_ and 4f_5/2_ at 158.8 and 164.1 eV, respectively) aside from two broad sidebands of the metallic core (Bi 4f_7/2_ and 4f_5/2_ at 157.6 and 162.7 eV, respectively). Both the locations of the peaks and their separation (≈5.3 eV) are consistent with those reported in the literature.^[^ ^51^, ^52^ ^]^ From Figure 2h, the Bi^3+^ peaks are predominant and the Bi^0^ peaks are suppressed because the depth of analysis for an XPS measurement is typically <10 nm.^[^ ^53^ ^]^ The same sample was further verified by the XRD spectrum, confirming the orientation and the high crystallinity of the as‐grown core‐shell heterojunction NWs on a Si substrate (Figure 2i). Achieving lasing in the UV region requires a high structural factor and thus a good internal quantum efficiency. The XRD pattern is indexed to a well‐crystallized rhombohedral Bi structure (Joint Committee on Powder Diffraction Standards No. 05–0519, R$\bar{3}$m), and it suggests a growth direction of the NWs with [0$\bar{1}$1] and [110] perpendicular to the substrate. Distinct XRD signals are observed, indicating the presence of lattice planes and hence a single crystalline phase, as supported also by the HRTEM investigation (Figure 2 and Figure S5, Supporting Information). These observations unambiguously confirm that the pristine Bi core provides the template for native oxidation, bridging the core and the outer β‐Bi_2_O_3_ shell together. The above results clearly provide direct information about the core‒shell heterojunction structure and the presence of quantum confinement in the sample.

### 2.2 Correlation between Photonics and Plasmonics among Core‒Shell Metal‒Semiconductor Heterojunction NWs

Most SPR‐assisted nanolasers have, so far, used exorbitant and scarce noble metals, such as gold (Au) or silver (Ag), which are not compatible with microelectronic manufacturing technologies. In this study, we focus on the use of cost‐effective Bi as an efficient UV scatterer because of its availability, facile synthesis, and microelectronic compatibility.^[^ ^13^, ^14^ ^]^ In fact, Bi has been the SPR material of choice for broad tunability across the UV and visible (VIS) spectral regions.^[^ ^15^, ^16^, ^17^, ^54^ ^]^

**Figure 3:**
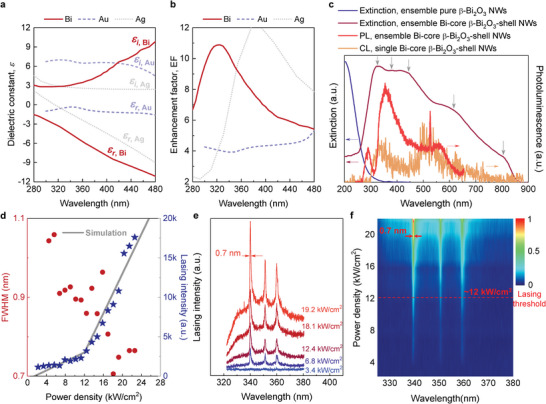
Spectroscopic characterizations and lasing performance of a core‒shell metal‒semiconductor heterojunction NW device. a) Theoretical dielectric functions of Bi, Au, and Ag, which were employed for the extracted enhancement factor, EF, in (b). *ε_i_* and *ε_r_* represent the real and the imaginary parts of the dielectric function, respectively. b) Predicted EF for the SPR in Bi, Au, and Ag, respectively, indicating the potential of Bi as an efficient SPR agent below ≈360 nm. c) Extinction, PL, and CL spectra of the demonstrated heterojunction NW device, shown alongside the extinction spectrum of pure β‐Bi_2_O_3_ shell for comparison. An overlap between the extinction (purple) and PL (red) spectra ensures efficient SPR coupling, leading to a strong interband transition for spontaneous emission and, thus, an enhanced stimulated emission. The main reason for slow modulations (denoted by the gray arrows) is given in the main text. d) Nanolaser performance with increasing excitation power density in linear scale clearly shows a threshold of ≈12 kW cm^−2^. The solid curve is a fit to rate equations (for details see Section S6, Supporting Information). e) Representative emission spectra below and above the lasing threshold. f) Pseudo‐color map of primary lasing peaks under various excitation levels, suggesting that the resonance modes were effectively amplified among the NWs, as detailed in Figure S11 (Supporting Information).

**Figure** **3**a shows the real (*ε_r_*) and the imaginary (*ε_i_*) parts of the dielectric function of metallic Bi, along with those of two widely used plasmonic materials, Au and Ag, for comparison. As seen in Figure 3b, it is important to point out that the dielectric response of Bi falls in the UV region (<360 nm), which leads to an enhancement factor, EF, of the SPR intensity to be higher than those of the noble Au and Ag. The enhancement factor is defined as^[^ ^55^ ^]^

(1) $$\mathrm{EF}=\frac{{\left|E\right|}^{2}}{{\left|E_{0}\right|}^{2}}={\left[\frac{2(\varepsilon (\omega )-1)}{\varepsilon (\omega )+2}\right]}^{2}=\frac{{\left(2\varepsilon_{r}-2\right)}^{2}+4\varepsilon_{i}^{2}}{{\left(\varepsilon_{r}+2\right)}^{2}+\varepsilon_{i}^{2}}$$

where |*E*_0_|^2^ is the intensity of an external field. As shown in Figure S7 (Supporting Information), Bi has a higher EF than those of common toxic and costly plasmonic metals of gallium and indium in the 300‒400 nm wavelength range because of its negative real dielectric response.^[^ ^42^, ^55^, ^56^, ^57^ ^]^ The literature values shown in Figure 3a for Bi, Au, and Ag are from Toudert,^[^ ^15^ ^]^ McPeak,^[^ ^58^ ^]^ and Werner,^[^ ^59^ ^]^ respectively. The parameters from Toudert in a Bi nanostructure were chosen as a reference throughout this work. We reasoned that those highly dense core‒shell NWs would create considerable plasmonic hot spots in the near field, which in turn would greatly promote sustained optical gain, as discussed in detail below.

Leveraging on the wide‐bandgap nature of β‐Bi_2_O_3_,^[^ ^60^, ^61^ ^]^ the heterojunction NWs could engender UV operation at wavelengths much shorter than those previously obtained from conventional GaN or ZnO materials. To study the contribution of the SPR from the Bi cores, one of the as‐grown samples (Bi core with β‐Bi_2_O_3_ shell) was intentionally treated in an oxygen condition and thereby was fully transformed into β‐Bi_2_O_3_ NWs for comparison, as confirmed by the Raman measurement in Figure S8 (Supporting Information). Figure 3c presents the extinction spectrum of pure β‐Bi_2_O_3_ NWs (blue), as well as the extinction (purple) and PL (red) spectra of our device (i.e., Bi‐β‐Bi_2_O_3_ core‒shell NWs); in the core‒shell structure, the β‐Bi_2_O_3_ shell serves as the gain medium in this study. From Figure 3c, the ensemble of pure β‐Bi_2_O_3_ NWs has an extinction spectrum peaked at ≈230 nm, corresponding to a bandgap energy (*E_g_*) of ≈4.4 eV (Figure S9, Supporting Information), analogous to the case prepared in a hydrothermal reaction (≈4 eV).^[^ ^61^ ^]^ Notably, the measured blueshifted bandgap of the pure β‐Bi_2_O_3_ NWs in comparison with that of bulk forms (≈2.3‒2.6 eV) seemingly reveals the effect of quantum confinement due to the small NW size,^[^ ^61^, ^62^, ^63^, ^64^, ^65^ ^]^ which is similar to gold and silver nanoforms.^[^ ^66^, ^67^, ^68^, ^69^ ^]^ The above result is supported also by the Raman investigation (Figure 2g). Further, the observed extinction spectrum of the as‐grown core‒shell heterojunction NWs exhibits a large broadening and a redshift (purple, Figure 3c), which also indicates that the ensemble Bi NWs are distinctly inhomogeneous in either geometry or orientation, as previously demonstrated in disordered plasmonic nanostructures.^[^ ^28^, ^70^, ^71^ ^]^ This feature can be inferred from both SEM and TEM images (Figures S1, S4, and S5, Supporting Information). Apparently, in our case, the significant orientation dispersion enables multicolored tuning of SPR over a broad UV‒VIS region rather than an ordered conformation.

The red curve in Figure 3c is a representative PL spectrum of ensemble heterojunction Bi NWs. This spectrum has a feeble emission band of a power density lower than 1 kW cm^−2^ at 290 nm, which is attributed to the near‐band‐edge transitions, and two broad and dominant luminescence peaks in the 325‒600 nm range originated from the trapping state and the electron‐hole recombination of band transition, respectively, as observed in β‐Bi_2_O_3_ nanoflowers and nanohooks.^[^ ^60^, ^72^, ^73^, ^74^, ^75^ ^]^ Meanwhile, the clear luminescence peaks at ≈340 and 510 nm in the cathodoluminescence (CL) spectrum of single heterojunction NW (orange, Figure 3c) are indicative of the PL spectrum (red, Figure 3c) of an ensemble of heterojunction NWs. Further, as summarized in Figure S10 (Supporting Information), the exemplary CL spectra of three individual NWs for the present sample were observed to vary from one NW to another (i.e., position‐dependent CL emissions); however, they exhibited a similar spectral feature, i.e., a board emission in the 300‒600 nm range. This also suggests that the measured PL of the device is the emission of an ensemble of Bi NWs.

Surprisingly, notwithstanding the previously reported broadband characteristic of UV‐excited PL spectrum,^[^ ^72^, ^73^, ^76^, ^77^, ^78^, ^79^ ^]^ the PL spectrum of our NWs for excitation at a power density higher than 1 kW cm^−2^ shows much more distinct cavity modes protruding at ≈340 nm rather than at ≈350 nm for excitation at a power density lower than 1 kW cm^−2^. We ascribe this blueshift to the band‐filling effect because the measured shift of the optical‐gain peak with excitation is in good accordance with the peak of the extinction spectrum at ≈340 nm (refer to Figure S11a, Supporting Information). The spectral widths of the optical‐gain peak in Figure S11b are found to be from 11 to 28 nm for the four cavity modes, which discretize the broad PL spectrum (Figure 3c). This feature indicates the effective cavity resonance established by densely packed core‒shell NWs, which provides the necessary feedback for lasing. The process is similar to the optical feedback via total internal reflection in a plasmonic laser of thin CdS nanosquares,^[^ ^80^ ^]^ as inferred from the mode selection addressed later.

Typically, periodic spectral fluctuations would occur in the extinction and PL spectra owing to the F‒P interference. In the extinction spectrum, distinct oscillation peaks were not found, but slowly modulated patterns were generated, as denoted by the gray arrows in Figure 3c. This is attributable to the tapered apexes with sharp corners relative to those with rounded structures.^[^ ^81^ ^]^ The extinction properties for a specified plasmonic nanostructure are significantly affected by the increase in the number of nanocones, as shown in the present study. Polarization dependence has been shown to modify the resonance signature of extinction responses.^[^ ^82^ ^]^ In our experiment, unpolarized white illumination was used, which may be another dominant factor that diminishes the oscillatory behavior.

Based on the guided PL spectrum shown in Figure S12 (bottom panel), the comparative spectral responses indicated consistency among several oscillatory peaks marked by the dashed lines. Because the direction of the polarized excitation field can be unrestrictedly rotated during the experiment, it was obtained by slightly adjusting the incident polarization. The spectral modulation in the guided PL spectrum would be further suppressed owing to the strong optical absorption of the metallic Bi core across the entire spectral region, as observed in the case of core‒shell NWs.^[^ ^83^ ^]^ Based on the metallic absorption‐loss spectrum shown in Figure S11b (Supporting Information), the linewidth broadening of the cavity modes reflects the energy loss within the device, i.e., the linewidth of the resonant cavity modes increases gradually with the emission wavelength, and the parasitic metallic loss exhibits the same trend.

In a core‒shell heterostructure, it is speculated by considering the large surface‐to‐volume ratio of core‒shell heterojunction NWs that the 290‐nm near‐band‐edge emission is governed by the quantum size effect because bulk β‐Bi_2_O_3_ crystals exhibit no PL.^[^ ^84^ ^]^ This result corroborates the aforementioned Raman spectra and blueshifted bandgap obtained from ensemble Bi‐core β‐Bi_2_O_3_‐shell NWs and pure β‐Bi_2_O_3_ NWs, respectively (Figure 2 g and Figure S8, Supporting Information). In the present case, strong UV scattering becomes prevalent as the PL spectrum is well within that of the SPR of the core‒shell heterojunction NWs. Figure 3c also clearly shows that the emitted photon energy of the β‐Bi_2_O_3_‐shell NWs at 350 nm is in satisfactory agreement with the SPR energy of the Bi core at 330 nm. Despite the fact that the optical loss in Bi metals is larger than that in β‐Bi_2_O_3_ semiconductors, a Bi metal core not only enhances plasmonic absorption but also facilitates the plasmonic gain, leading to an overall resonance‐dependent net gain.^[^ ^85^ ^]^ Based on this result, as of concern to a potential plasmonic nanolaser, it is expected that the strong SPR coupling allows an interband transition and further readies band‐to‐band recombination for efficient spontaneous and stimulated emissions from the core‒shell heterojunction NWs.

### 2.3 UV Low‐Threshold Lasing in a Photonic‒Plasmonic‐Coupled NW Device and its Underlying Mechanism

In conjunction with the VS technique endowed with inherently easy handling, and high yield yet high crystallinity, we demonstrate that achieving the densely disordered NWs and optically flat F‒P microcavity formed by a naturally formed metal‒semiconductor heterostructure. These NWs represent a significant step toward the SPR‐coupled nanoemitter. The photonic‒plasmonic‐coupled NW ensemble is optically pumped by a 263‐nm nanosecond‐pulsed laser at RT (Figure S3, Supporting Information). Figure 3d shows that the integrated output intensity and the full width at half‐maximum (FWHM) of its spectrum against the excitation power density follow a nonlinear response, delineating clear features of the onset of lasing. We spectroscopically observed an FWHM reduction from the typical broad spectral width of the PL down to ≈0.7 nm when the excitation power density was increased, as observed for the disordered nanolaser analogy.^[^ ^86^ ^]^ This characteristic demonstrates that the cavity modes are selectively amplified by optical feedback in the dense heterojunction NW cavity. Importantly, Figure S13 (Supporting Information) shows the interference fringes above the laser threshold; furthermore, it clearly indicates the onset of lasing. Figure S14 (Supporting Information) shows a photograph of the far‐field pattern of the lasing output, where a sensor card was placed 30 cm in front of the nanolaser device. The high directionality and coherence of the device were demonstrated.

The representative emission spectra for different excitation power densities below and above the lasing threshold are shown in Figure 3e. Nearly equidistant longitudinal modes at 340.1, 350.1, and 360.0 nm can be clearly resolved. At an excitation level above 12 kW cm^−2^, the emission remarkably intensifies, and the mode at 340.1 nm stands out over the broad spontaneous emission band with a significantly reduced FWHM down to ≈0.7 nm. (The observed lasing linewidths of ≈0.7 nm were limited by the resolution of the detection system.) Thus, the lasing threshold is determined to be at 12 kW cm^−2^. Above the lasing threshold, the FWHMs of the three adjacent lasing modes are all below 0.8 nm (Figure S15), far narrower than that of the broad PL spectrum. The narrowed linewidths are comparable with the previously reported RT UV lasers (Tables S1‒S3, Supporting Information), confirming that the observed sharp spectral peaks represent UV lasing. It should be noted that the f‐element lanthanides typically result in a set of narrow lines. However, severe lanthanide‐related signals would occur in both the XPS survey scan (Figure S2, Supporting Information) and Raman spectrum (Figure 2g) if they were included in this study. XPS and Raman analyses can provide direct elemental information regarding the presence of unintentionally introduced dopants owing to their high quantitative and qualitative sensitivities. Notably, we have never performed lanthanide‐based experiments previously; hence, contamination can be precluded.

Moreover, from the SEM image in Figure 1c, the cavity length *L* is ≈6.1 µm; therefore, the corresponding lasing wavelength *λ*_L_ for the lasing mode can be theoretically predicted by introducing the mode spacing △*λ* and the effective refractive index *n* into the classical F‒P cavity model: *λ*_L_^2^ = △*λ*{2*L*[*n*‐*λ*_L_(*dn*/*dλ*_L_)]}. The effective *n* is estimated by considering both the core‐to‐shell volume fraction (≈70%) and the filling factor (≈17.5%). Figure S16 (Supporting Information) presents the corresponding dispersion relation. By taking △*λ*, *L*, and *n* to have the values of 10 nm, 6.1 µm, and 1.23, respectively, the value of *λ*_L_ is found to be ≈349.3 nm, which is close to the experimentally observed value of 350.1 nm determined from the lasing spectrum shown in Figure 3e, thus verifying that the laser action arises from a vertical F‒P microcavity. Furthermore, the presence of a series of narrow lasing peaks also indicates that the top and bottom end faces of the densely packed NWs effectively act as two partial mirrors for the F‒P microcavity.^[^ ^87^ ^]^

By using our proposed technique, high‐quality Bi NWs can be facilely grown, with a precisely controlled thickness, on a Si substrate. Although some of the Bi NWs protruded from the surface of the ensemble NWs (Figure 1c), we discovered that they minimally affected the resonance when the amount of protrusion in the present sample was significantly lower than that in the ensemble counterpart. On the other hand, because the free spectral range is governed by the thickness of the Bi NWs, the variation in the lasing peaks is attributable to the thickness‐dependent optical properties. However, we observed the lasing behavior only in the sample with an average thickness of ≈6.1 µm. One important parameter that determines the laser action is the net optical gain of the device, which is related not only to the enhanced plasmonic gain but also to the metallic absorption loss. For a thinner sample, the most intuitive conclusion may be a reduction in the hot‐spot densities when the thickness decreases, which consequently results in an insufficient plasmonic optical gain. By contrast, as the sample thickness increases, the metallic core volume increases significantly within the device, thus resulting in a high metallic absorption loss. This may be related to the absence of laser action in a thicker sample.

**Figure 4:**
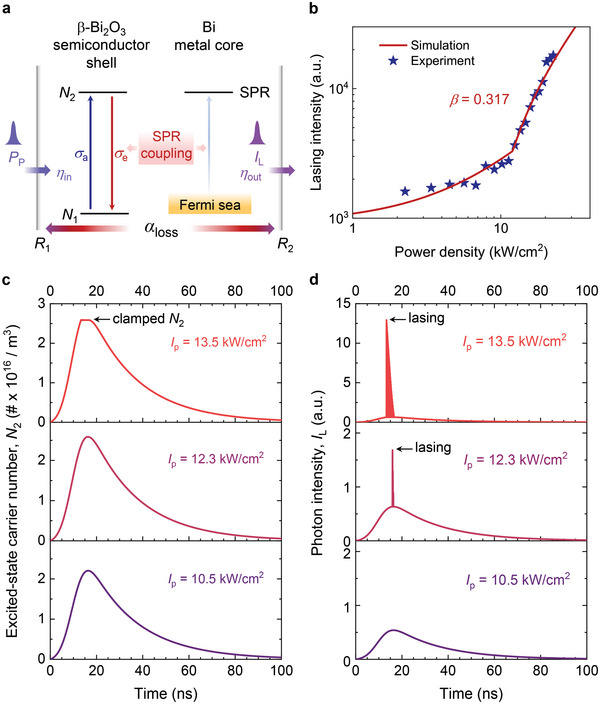
Temporal dynamics of the hybrid photonic‒plasmonic‐coupled F‒P nanolaser. a) Schematic of the resonant intermodal coupling between the photonic cavity modes and the SPR‐enhanced modes. b) Dependence of the lasing intensity on the incident power density, the same data presented in Figure 3d. The solid curve was simulated for the experiment using a coupled time‐dependent model having a high *β* factor of 0.317. c,d) Turn‐on lasing dynamics of the excited‐state carrier number (*N*_2_) and the photon intensity (*I*_L_), showing the transient state from spontaneous emission to stimulated emission.

Yet, at this stage, the typical photonic model is inadequate for explaining the observed narrow lasing linewidths on account of the low reflectivities at both the air/NWs and the NWs/Si‐substrate interfaces, which could be alternatively attributed to the enhanced light‒matter interactions within the core‒shell heterojunctions, as further discussed below. Figure 3f presents the representative spectral map as a function of the excitation power density. It is apparent that the lasing peaks are fixed and independent of the excitation level above the threshold. This result supplies further evidence that the UV lasing does not stem from the resonant feedback in a closed‐loop light‐scattering manner, as usually found for random schemes.^[^ ^88^, ^89^, ^90^ ^]^ Having demonstrated that UV lasing in naturally formed heterojunction NWs and light confinement in the vertical microcavity was well interpreted by the F‒P model, here we raise another question: it is intriguing to speculate whether the aforementioned lasing behavior only originates from general F‒P resonance. Remarkably, on the basis of a rate‐equation model (see **Figure** **4**a and Section S6, Supporting Information), the theoretical fit and the measured data agree well (Figures 3d and 4b), manifesting the low‐threshold UV lasing from the synergistic photonic‒plasmonic coupling, as elaborated in detail below. Note that the metallic absorption‐loss spectrum (Figure S11b, Supporting Information) shows considerable energy losses across the entire spectral region. This implies that whereas light is incident on the test samples, the PL emission is relatively weak at excitation intensities below 1 kW cm^−2^, as similarly indicated by the *x*‐axis intercept of both the simulation and experiment.

Meanwhile, an analysis of Figure 3d yields a high coupling factor of *β* ≈0.32 (Figure S17 in Supporting Information). This is also evidenced by the temporal development of laser spiking associated with relaxation oscillations, in which the turn‐on surge emerges as the round‐trip gain overcomes the total round‐trip loss (Figure 4c,d). It is also noteworthy that the lasing threshold is as low as 12 kW cm^−2^, which is the lowest ever reported among nanolasers operated either at RT or at a wavelength shorter than 360 nm (Tables S1‒S3, Supporting Information). Furthermore, the output intensity of the same device against the input power density was tested again after one year, as shown in Figure S18a (Supporting Information). Although the lasing threshold was slightly increased to ≈13 kW cm^−2^, in conjunction with the spectral evolution shown in Figure S18b (Supporting Information), the strong emission clearly indicates the robust reliability of our NW device and that of the demonstrated VS technique for mass production.

**Figure 5:**
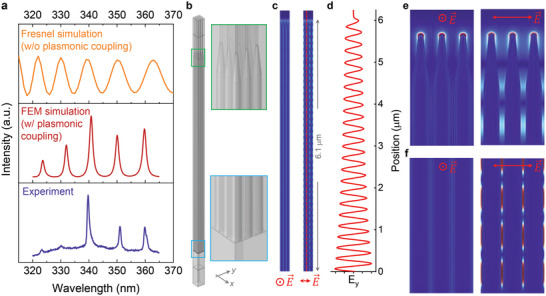
Simulated spectral and spatial responses of the hybrid photonic‒plasmonic‐coupled F‒P laser action. a) Top panel: plot of spectral response calculated by Equation (2) showing a rather broad modulation due to multiple interferences at each interface, as expected from the classical Fresnel relation. Center panel: FEM simulated spectrum with plasmonic coupling, showing a series of narrow resonances with the F‒P characteristics in good agreement with the experimental spectrum given in the bottom panel. Bottom panel: Experimentally recorded lasing spectrum. b) Computational model reconstructed from a realistic NW geometry based on SEM and HRTEM images. c) Spatial *E*‐field maps of (b) for in‐plane and out‐of‐plane polarization. d) Plot of the *E* field between the two adjoining NWs for a slice of the *E*‐field intensity (red dashed line in (c)) from the top to the bottom. e,f) Enlargements of (c) on the top and the bottom of the NWs, witnessing the synergy of localized SPR and the significant standing‐wave character. Blue represents zero *E* field; red max *E* field.

Given the exceptionally low‐threshold and high‐*β* lasing at RT, we then delved deeper into the effect of the photon‒plasmon coupling strength on the laser action and its underlying mechanism. Phenomenologically, we simulated the intracavity properties without and with involving plasmonic couplings to unravel their spectral responses. In this regard, the corresponding enhanced effective reflectivity was thus obtained. The spectrum in the top panel of **Figure** **5**a was determined by the classical F‒P model (Equation (2)) without plasmonic coupling, whereas that in the center panel of Figure 5a was calculated by using the finite element method (FEM) via a variable frequency to excite the hybrid photonic‒plasmonic modes (details refer to Section S4, Supporting Information). Note that, due to the intrinsically dense nature of the core‒shell heterojunction NWs, one can reasonably simplify the present form, comprising of densely‐packed NWs standing on a thin supporting Bi/Bi_2_O_3_ interlayer, as an F‒P thin film on a Si substrate, as schematically illustrated in Figure 1a. Herein, the intracavity spectral response *I* due to interference within the thin film of a thickness *d* can be extracted based on the known relation in terms of the intensity of the incident wave *I*_0_, the reflectivities *R*_1_ and *R*_2_ at the air/NWs and NWs/Si‐substrate interfaces, respectively, and the refractive index *n*, through the longitudinal mode function:^[^ ^91^ ^]^

(2) $$\frac{I_{0}}{I}={\left(1-\sqrt{R_{1}R_{2}}\right)}^{2}+4\sqrt{R_{1}R_{2}}\sin^{2}\left(\pi nd/\lambda \right)$$

In the above expression, the employed values of *I*_0_, *R*_1_, *R*_2_, *n*, and *d* for the core‒shell heterojunction NWs were 1, 1.03%, 38.48%, 1.23, and 6.1 µm, respectively. Even though the oscillatory characteristics in the top panel of Figure 5a exhibit strong modulations and the positions of maximum intensities correspond well to those of the experimentally observed lasing peaks (bottom panel, Figure 5a), its rather broad undulating FWHMs do not support the result of narrowed lasing linewidths. The narrowed linewidth features multiple interferences within a thin layer with two partially reflective mirrors formed by Fresnel reflections. Undoubtedly, the low reflectivities, *R*_1_ (≈1.03%) and *R*_2_ (≈38.48%), of the two interfaces are not able to effectively sustain the optical resonance, particularly for the stimulated emission, which indicates that there are other mechanisms involved in the UV laser action.

To further shed light on the contribution of the photonic‒plasmonic‐coupled lasing mode, we performed 2D FEM simulations to determine the spectral response (center panel, Figure 5a) and the corresponding spatial map of the electric field (*E* field) in both the *xz* and *yz* planes (Figure 5b–f). This model was based on a realistic representation (i.e., the average thickness, diameter, and spacing) of the core‒shell nanostructures and the bottom supporting Bi/Bi_2_O_3_ interlayer and was constructed by identifying the morphology and geometry found in the aforementioned SEM and HRTEM images, respectively. As seen in Figure 5a, the agreement between the numerically simulated (center panel) and the experimentally observed (bottom panel) spectra is excellent. This conspicuous spectral correspondence in Figure 5a unambiguously manifests that the SPs indeed play a key role in the low‐threshold RT operation of the UV laser action. Note that to qualitatively reproduce the plasmonic properties in a randomly distributed manner, the hot‐spot features should be modeled in 3D space. However, 3D disordered finite element modeling was not performed owing to the significant amount of time and the corresponding computational burden required for solving 3D geometries. Hence, a 2D periodically ordered model was used to model densely packed Bi NWs. Our fabrication strategy indicated a 100‐fold improvement in growth density as compared with previous strategies. As mentioned earlier, the present form comprising densely packed NWs can be reasonably simplified to an F‒P thin film on a Si substrate. Additionally, the effective cavity resonance established by densely packed core‒shell NWs provides the necessary feedback for lasing. Meanwhile, the spectral agreement shown in Figure 5a may be regarded as further support for validating the abovementioned F‒P thin‐film approximation.

A simulated spatial map of the *E* field among the NWs, which is shown alongside that for a slice of the *E*‐field intensity as a function of the position in Figure 5d, is shown in Figure 5c. The result presents a reasonable mimic of intermodal coupling, predicting many of its intriguing features. Particularly, the photonic‒plasmonic‐coupled resonances among the NWs have primarily a standing‐wave characteristic with much of the *E* field localized in the periphery of the shell, as evidenced in the magnified images of Figure 5e. suggests the existence of centered hot spots near the apex of the NWs because nanostructures with sharp cones promote charge separation more easily than smooth shapes. It is clear that there are pronounced *E*‐field enhancements on the top (Figure 5e) and the bottom (Figure 5f) of the NWs owing to the unique tapered nanostructures.^[^ ^92^, ^93^, ^94^ ^]^ Meanwhile, the randomness of the orientally tapered NWs provides a variety of diameters, and it efficiently traps incoming photons and transmits them down to the substrate,^[^ ^95^, ^96^, ^97^, ^98^, ^99^ ^]^ as implied by the broadband extinction spectrum presented in Figure 3c. Note that Figure 5e depicts further evidence that the tapered apexes function as nanoantennae and thus facilitate the coupling of incident light into SPR at the Bi/Bi_2_O_3_ heterojunction interface. Actually, the core‒shell metal‒semiconductor heterojunction is expected to intensify the light‒matter interplays because of the extent of hybrid coupling between the photons (i.e., photonics) and the SPR (i.e., plasmonics).^[^ ^100^ ^]^ In this connection, the gain layer consists primarily of densely distributed nanometer‐sized apexes, leading to the generation of a substantial amount of the strong and localized *E* fields across the whole F‒P microcavity and hence the SPR‐assisted UV laser action.

From the center panel in Figure 5a, we see that there are a considerable number of longitudinal modes within the gain bandwidth. A key factor is the wavelength range of the mode selection in relation to that of the parasitic metallic loss (Sections S7 and S9, Supporting Information). This is particularly clear as one looks into the absorption and emission cross‐sections. The oscillatory laser modes are determined by the optical‐gain spectra and the parasitic metallic loss. (Figure S19 and Section S10, Supporting Information). If a series of sharp emission lines were due solely to F‒P resonance involving an emission cross‐section that is of the order of 10^−13^ m^2^, then the optical gain of the material is not able to overcome the intrinsic metallic loss across a broadband span (Figure S19a), suggesting no laser action. This is not the case in reality. A refined calculation based on coupled rate equations in which the emission cross‐section is modified by a 100‐fold enhancement highlights the requirement for adequate net gain (Figure S19b). This result rationalizes the fact that the SPR plays a key role in intensifying the effective amplification and stimulated emission within the NW microcavity.

We now turn to consider the SPR‐enhanced effects on the effective cavity reflectivities, *R*, in our system. For our device, an analytical expression in terms of the quality factor and the parasitic metallic absorption loss at the lasing wavelength enables us to clarify the physical parameters in sustaining the stimulated emission (Section S11, Supporting Information). From the derived *R*‐value of ≈75.9%, it becomes evident that the SPR effect is expected to significantly enhance the reflectivity. The increased *R* can then overcome the intrinsically high metallic loss and the low modal reflectivity, thereby achieving a low‐threshold UV nanolaser. The developed nanolaser can serve as a compact and cost‐effective alternative to either bulky solid‐state or gas UV lasers emitting below 360 nm for bioanalytical applications.^[^ ^101^, ^102^, ^103^ ^]^ Our proposed simple yet high‐yield vapor‒solid technique does not involve expensive prerequisites but enables a scalable yet low‐cost approach for developing compact integrated devices. This technique is practical for a wide range of metal‒semiconductor heterojunction NWs. Thus, by adopting plasmonic aluminium,^[^ ^58^ ^]^ indium,^[^ ^55^ ^]^ or Ag,^[^ ^104^ ^]^ we expect on‐chip low‐threshold heterojunction nanolasers for UV‐B (280‒320 nm), UV‐C (< 280 nm), or VIS/near‐infrared bands to be realizable on Si substrates.

## 3 Conclusion

In summary, we have experimentally demonstrated and theoretically studied the core‒shell metal‒semiconductor heterojunction NWs that are grown directly on a large‐area Si substrate as a route toward a compact and low‐threshold UV nanolaser. To fully meet the requirements of facile and scalable fabrication, each of the densely packed and optically flat metallic Bi cores of a high crystallinity was grown with a self‐terminating Bi_2_O_3_ native semiconductor shell, thus forming a vertical F‒P microcavity for the circulation of light through effective multiple reflections in each NW. Unlike most single‐NW lasers that lase after they are removed from the substrate, we show that direct lasing of the ensemble NWs on a Si substrate with enhanced Fresnel reflection between two opposing parallel crystalline facets is possible because the densely packed ensemble NWs that form the gain medium also serve as an efficient F‒P resonator for the formation of cavity modes. Additionally, the good overlap between the SPR band and the PL spectrum of the optical gain material delineates beneficial SPR‐field enhancement that underpins the PL enhancement by up to 100 times. In light of this, through the intermodal coupling between the photonic cavity modes and the plasmonic resonance modes, UV lasing with a record‐low threshold and a high‐*β* factor were successfully achieved. The results presented in this paper demonstrate the promising prospects of the proposed heterojunction platform for the integration of active optical devices in silicon‐based nanophotonic systems.

## Conflict of Interest

The authors declare no conflict of interest.

## Supporting information

Supporting Information

## Data Availability

The data that support the findings of this study are available in the supplementary material of this article.
