# Supplementary material for: On‐Chip Monolithically Integrated Ultraviolet Low‐Threshold Plasmonic Metal‒Semiconductor Heterojunction Nanolasers
Source: Adv Sci (Weinh). 2023 Aug 9;10(28):2301493. doi: 10.1002/advs.202301493 (PMC10558691; doi:10.1002/advs.202301493)
Supplement: Supplementary file 1 — Supporting Information [file ADVS-10-2301493-s001.pdf]

ADVANCED SCIENCE
Open Access

Supporting Information

for *Adv. Sci.*, DOI 10.1002/advs.202301493

On-Chip Monolithically Integrated Ultraviolet Low-Threshold Plasmonic Metal–Semiconductor Heterojunction Nanolasers

*Jia-Yuan Sun, Duc Huy Nguyen, Jia-Ming Liu, Chia-Yao Lo, Yuan-Ron Ma, Yi-Jia Chen, Jui-Yun Yi, Jian-Zhi Huang, Hien Giap, Hai Yen Thi Nguyen, Chun-Da Liao, Ming-Yi Lin and Chien-Chih Lai**

Copyright 2023. Wiley-VCH GmbH.

Supporting Information

On-Chip Monolithically Integrated Ultraviolet Low-Threshold Plasmonic Metal-Semiconductor Heterojunction Nanolasers

*Jia-Yuan Sun[1,11], Duc Huy Nguyen[1,11], Jia-Ming Liu[2,3,4], Chia-Yao Lo[5], Yuan-Ron Ma[1], Yi-Jia Chen[6], Jui-Yun Yi[7], Jian-Zhi Huang[8], Hien Giap[1], Hai Yen Thi Nguyen[1], Chun-Da Liao[9], Ming-Yi Lin[10], and Chien-Chih Lai[1,8]**

[1]Department of Physics, National Dong Hwa University, Hualien, 974301, Taiwan

[2]Department of Electrical and Computer Engineering, University of California, Los Angeles, CA 90095, USA

[3]Institute of Photonics, National Yang Ming Chiao Tung University, Tainan 711010, Taiwan

[4]Institute of Optoelectronics, National Chung Hsing University, Taichung 402202, Taiwan

[5]Department of Optoelectronics and Materials Technology, National Taiwan Ocean University, Keelung 202301, Taiwan

[6]Department of Materials Science and Engineering, National Dong Hwa University, Hualien 974301, Taiwan

[7]Department of Electrical Engineering, National Kaohsiung Normal University, Kaohsiung 824004, Taiwan

[8]Department of Opto-Electronic Engineering, National Dong Hwa University, Hualien 974301, Taiwan

[9]R&D Center, Taiwan Semiconductor Manufacturing Company, Hsinchu 300091, Taiwan

[10]Department of Dermatology, National Taiwan University Hospital and College of Medicine, National Taiwan University, Taipei 100229, Taiwan

[11]These authors contributed equally: Jia-Yuan Sun, Duc Huy Nguyen

*E-mail: cclai@gms.ndhu.edu.tw

Methods

Section S1, Fabrication of the core–shell metal–semiconductor heterojunction NW:

To utilize the benefits of simple and low-cost fabrications, dense Bi nanowires (NWs) contact with silicon <100> substrate was grown against 10^{-4} torr at a temperature range of 800–900 °C using a home-built vapor–solid (VS) system. This offers an even higher heating and cooling rate (>100 °C/s); hence a significant internal compressive stress to form smaller and denser nanograins. The VS-based technique is favored owing to the simplicity and flexibility in producing various single crystalline structure with high quality.[1–7] The purity of 99.99% Bi, having an average ingot size of approximately 1 mm, was used as the raw material. The deposition process was implemented by heating the resistive tungsten plate to sublime the dispersed Bi ingots and deposition of thin Bi films on the bottom surface of the top Si substrate, as schematized in Figure S1.

A hollow graphite cylinder separated a small distance between the top substrate and the bottom plate. The source-to-substrate distance was set at 2 mm, which prevents time-consuming processes and fosters fast evaporation, and promotes a high material utilization ratio because the deposition rate is inversely proportional to the second power of the spacing distance. The high growth temperature and rapid cooling play a key role in providing a large compressive stress that facilitates a high yield rate of Bi NWs.[8–15] By exposing to an oxygen-rich environment, Bi NWs form a thin, self-terminating Bi_2O_3 shell that preserves the metallic Bi core. The samples were stored under ambient conditions for over one year, devoid of any special treatment, demonstrating the effective robustness of the thin Bi_2O_3 shell. For device demonstration, silicon was chosen as the substrate because of the importance of making optical devices mechanically flexible and robust, fostering improved heat dissipation, and enabling a stronger damage threshold. The silicon surface was carefully cleaned before use to remove adventitious organic contaminants. Instead, although natively formed SiO_2 layers are present on the Si surface, their effect is negligible because the thickness (a few to tens of nanometers) is far behind the wavelength of ultraviolet (UV) photons as the light travels through it.

Section S2, Structural characterization:

Diversified electron microscopy and X-ray microscopy were used to determine the dimensions, growth morphology, and local composition. Two field-emission scanning electron microscopes (SEMs) were applied for high- (Helios NanoLab G3 CX, FEI) and low-magnification (JSM-7000F, JEOL) inspections. Atomic-scale investigations were performed using a field-emission

high-resolution transmission electron microscope (TEM; Tecnai G^2 F20, FEI) operating at an acceleration voltage of 120 kV. All the TEM samples were prepared using a focus ion beam (FIB; NX2000, Hitachi) system. Compositional information was acquired using an X-ray photoelectron spectroscope (XPS; K-Alpha, Thermo Scientific). The optical properties of the individual core–shell metal–semiconductor heterojunction NW were measured by room-temperature (RT) cathodoluminescence (CL) using a Gatan Mono-CL system attached to the SEM (JSM-7001, JEOL). For charge correction across the wide XPS survey scans (Figure S2), the standard reference of the C 1s (284.5 eV) signal was verified before applying it to all XPS peaks in Bi and O elements. In addition, X-ray diffraction (XRD) combined with selected-area electron diffraction patterns from high-resolution TEM were employed to identify the crystallographic relationship between the Bi NW core and the Si substrate. XRD was conducted using a diffractometer (X'Pert Pro, PANalytical) with Cu Kα radiation set at 1.54056 Å, with a scan step of 0.02° and a dwell time of 90 s at RT.

Section S3, Optical characterization:

We used a home-built multiwavelength laser confocal microscope for spatially and spectrally resolved micro-PL, micro-Raman, and lasing measurements The NWs under test were probed vertically using continuous-wave and pulsed excitations, as presented in Figure S3. Raman spectroscopy was performed at 633 nm (N-LHP-925, Newport), while the PL and NW lasing behaviors were characterized at 263 nm (DTL-389QT, Laser-Export). Two objectives for micro-PL and micro-Raman were employed in the confocal microscope: 40× (LMU-40X-UVB, Thorlabs) and 100× (MPlanAPO, Olympus) to focus the laser and collect the backward signal from the excitation area. In our experiment, we installed a band-pass filter in front of the 263-nm excitation source to block the light from the other emission lines, including the third harmonic generated from the Nd:YAG source. The incident pump power was initially passed through an isolator and tuned using a calibrated half-wave plate and polarizer. The output emission images were captured by using a camera-based beam profiler (LT665, Ophir). All optical signals were gathered and guided by an optical fiber (PCN200-2-SS, Multimode Inc.) to a spectrometer equipped with a thermos-electrically cooled charge-coupled detector (DV420A-BU2, Andor). All spectra are exhibited as raw data; no smoothing has been processed, and all the optical experiments were performed at RT.

Section S4, Numerical calculation:

A rigorous finite-element-method model based on solving full-field Maxwell's equations with boundary conditions was adopted to explain the impact of SPR-coupled Fabry–Pérot (F–P) resonance modes and the electric-field (*E*-field) distribution. A 2D periodic FEM model was constructed by referring to the nanostructures (the length, diameter, and spacing) of hundreds of Bi NWs inspected directly from SEM and TEM images. On average, the employed length, diameter, and spacing were 6.1 μm, 65 nm, and 10 nm, respectively. All electrodynamics calculations were performed using periodic boundary conditions along the *x*- and the *y*- axes. The computational domain includes nine unit cells of the core–shell metal–semiconductor NW with rectangular dimensions of 70 nm × 70 nm × 6500 nm, as shown in Figure 5b. In this case, the Si substrate is extended to the edge of the computational domain. To reduce the simulation cost without sacrificing accuracy, a graded mesh space was discretized by a step of 7 and 1 nm within the core and the shell regions, respectively, by a refined dimension of 0.7 nm near the NW apex. Away from the NW, the modeling domain with an increased mesh step of 10 nm was truncated by a perfectly matched layer boundary condition for 3D electromagnetics to minimize the interior reflections over every incident angle. The *E*-field map of the SPR modes upon excitation with an incident plane wave was generated by evaluating the scattered *E*-field around the core–shell metal–semiconductor nanostructure. The overall resonance spectrum of the SPR-coupled F–P microcavity was acquired by integrating the stored energy over an enclosed volume. By applying these parameters, the simulation results agreed well with the experimental data, thus demonstrating the effectiveness of our proposed FEM model.

Section S5, Estimation of the laser focus spot:

The excitation spot sizes with radii *w* of 263 and 633 nm on the samples can be estimated by the Gaussian beam relation, given by $\lambda f(\pi w_0)^{-1}$ where λ, w_0, f are the excitation wavelength, the beam waist, and the focal length of the adopted objective, respectively. We used 263 nm, 0.5 mm, and 5 mm; 633 nm, 0.96 mm, and 0.74 mm for λ, w_0, and f, respectively. The resulting *w* values at 263 and 633 nm were approximately 837 and 155 nm, respectively.

Section S6, Simulation of the photonic–plasmonic-coupled heterojunction NW laser:

To delve into the lasing behavior, we adopted a rate equation analysis via time-dependent dynamics describing the coupled excited-state carrier number, $N_2(t)$, and the photon intensity, $I_L(t)$, as shown in Figures 4c and 4d, that is[16,17]

$$\frac{dN_2(t)}{dt}=\frac{\sigma_a\lambda_P I_P(t)}{hc}N_1(t)-\frac{\sigma_e\lambda_L I_L(t)}{hc}N_2(t)-\frac{N_2(t)}{\tau}, \tag{S1}$$

$$\frac{dI_L(t)}{dt}=I_L(t)\left[\frac{c}{n}\sigma_e N_2(t)-\frac{\beta}{t_c}\right], \tag{S2}$$

where the pump intensity $I_P(t)$, the pump term $P(t)$, and the photon decay rate t_c are given by

$$I_P(t)=\frac{P_P(t)\eta_{in}[\exp(-\alpha_{loss}^{P}d)][1-\exp(-\alpha_{abs}d)]}{\pi r^2}, \tag{S3}$$

$$P_P(t)=P_0\exp[-t^2/2\Delta t^2], \tag{S4}$$

and

$$t_c=\frac{2nd}{c\{1-[R_1R_2\exp(-\alpha_{loss}^{L}d)]\}}. \tag{S5}$$

In the preceding relations, λ_P, λ_L, N_1, h, and c are the pump and the lasing wavelengths, the ground-state carrier number, the Planck's constant, and the speed of light in a vacuum, respectively. σ_a, σ_e, τ are the absorption cross section at λ_P, the emission cross section at λ_L, and the spontaneous lifetime, respectively. n and β are the refractive index and the spontaneous coupling factor, respectively. n is related to its core-to-shell volume ratio $F\approx 70\%$ and average filling factor $F'\approx 17.5\%$, with the refractive indices of Bi (n_{Bi})[18] and Bi_2O_3 ($n_{Bi_2O_3}$)[19] given by $n=[Fn_{Bi}+(1-F)n_{Bi_2O_3}]\times F'+n_{air}\times(1\text{-}F')$, as shown in Figure S16. (Note, both the F and the F' were estimated by verifying the geometry and quantity based on SEM and HRTEM images. Specifically, more than 20 SEM images and 10 HRTEM images were scrutinized.) η_{in}, α_{abs}, d, and r are the coupling efficiency at the input end, the absorption coefficient at λ_P, the microcavity length, and the mode radius, respectively. R_1 and R_2 are the input and the output coupler reflectances, respectively. (One can reasonably calculate the normal incidence reflectivity by accounting for the corresponding n. For the given air/NW and NW/Si facets, R_1 and R_2 are approximately 1.03% and 38.48%, respectively.) P_0 and Δt are the peak power and the pulse width, respectively, where the full width of the pulse at e^{-1} intensity is $2\sqrt{2}\Delta t$. Such a model, in which the plasmonic effects of the gain material are considered by introducing parasitic metallic losses at λ_P (α_{loss}^{P}) and at λ_L (α_{loss}^{L}), can adequately describe the interplay among the transition, the population inversion, and the loss compensation.

The laser output is then related to its coupling efficiency, η_{out}, at the output end by $\eta_{out} I_L \pi r^2 (1 - R_2)/2$; and the absorption coefficient value at λ_P (α_{abs}) is based on the pump saturation intensity (I_{sat}) and the small-signal absorption coefficient (α_0).

$$\alpha_{abs} = \frac{\alpha_0}{1 + P_P(t)\eta_{in} \exp(-\alpha_{loss}^{P} d)/I_{sat}}, \tag{S6}$$

where $I_{sat}=(hc)/(\sigma_a \lambda_P \tau)$. The propagation losses, α_{prop}, at both λ_P and λ_L are negligible in view of the micrometer-sized cavity length because α_{prop} depends explicitly on the distributed loss per round trip, where $\exp(-\alpha_{prop}d)\approx 1$. The comparative data between the simulation results and the referenced parameters are summarized in Table S4. We input these parameters into the simulation model and found that the β factor and the laser threshold agreed well with the experimental values. In addition, we have examined the β factor based on Reference [20] (Supporting Information), where the authors postulated that the β factor can be directly determined from the ratio of the output intensity above and below the laser threshold, as indicated in the inset in Figure S17b. As shown in Figure S17a, the experimental data in Figure 3d can be well fitted with the rate equations considering β=0.317, showing a good correspondence of β=0.316 obtained by an analytical model (Figure S17b).[20] This correspondence verifies the effectiveness of our proposed laser simulation.

Section S7, Estimation of the absorption loss in Bi:

The light absorption induced by the Bi core at any wavelength ($\alpha_{loss}(\lambda)$), which results in significant optical losses that would severely deplete both the incident pump power and the intracavity photon intensity, must be considered in any case with the metal-semiconductor heterojunction, as formulated in Equations (S3) and (S5). Thus, α_{loss}^{P} and α_{loss}^{L} can be calculated from the real (ε_r) and the imaginary (ε_i) parts of the permittivity of Bi metal by[21]

$$\alpha_{loss}(\lambda) = \frac{4\pi\kappa(\lambda)}{\lambda}, \tag{S7}$$

where

$$\kappa(\lambda) = \frac{1}{\sqrt{2}}\sqrt{-\varepsilon_r(\lambda) + \sqrt{\varepsilon_r^2(\lambda) + \varepsilon_i^2(\lambda)}}\ . \tag{S8}$$

By substituting the values for ε_r and ε_i at λ_P and λ_L (Figure 3a), the resulting α_{loss}^{P} and α_{loss}^{L} values were approximately 7.33×10^7 and 8.08×10^7 m^{-1}, respectively. Meanwhile, considering an average filling factor (approximately 17.5%) along with a core-to-shell volume ratio (approximately 70%), this leads to a predicted α_{loss}^{P} and α_{loss}^{L} of approximately 8.98×10^6 and

approximately 9.90×10^{6} m^{-1}, respectively. These results agree with the numerically determined values of 5.14×10^{6} and 9.00×10^{6} m^{-1} for the λ_P and λ_L. The filling factor in influencing the metallic absorption loss and hence the population inversion for plasmonic nanolasers is reminiscent of the one observed for the randomly distributed AlGaN-NW case,[22] which suggests that the realization of efficient laser emission is, as expected, dominated by the size, the filling fraction, and the absorption loss of the plasmonic nanostructures.

Discussion

Section S8, Growth mechanism of the core–shell metal–semiconductor heterojunction NW:

The attractive fabrication routes for NW are based on catalyst-free (or template-free) VS systems through source heating and sublimation, nucleation, and crystallization of target materials. Apart from the commonly used physical vapor transport, which is performed with a high vacuum, inert gases, or even a bulky chamber, the proposed and demonstrated VS platform requires none. In addition to the aforementioned growth conditions, the parameter that most significantly influences the NW quality, density, and morphology is the tiny millimeter-scale spacing between the starting ingots and the top target substrate. Sublimation in our VS system can be predicted when the mean free path is much longer than the characteristic length. From the growth temperature (T) and the pressure (P), the mean free path (λ_{mean}) of Bi vapor at the macroscale can be directly calculated from the Maxwell–Boltzmann distribution relation[23]

$$\lambda_{\text{mean}} = \frac{k_B T}{\sqrt{2}\pi D^2 P}, \tag{S9}$$

where k_B is the Boltzmann constant, and D is the Van der Waals diameter of the Bi atom.

Considering the contribution of k_B=1.38×10^{-23} J·s·K^{-1}, T=1173 K, D=2.07×10^{-10} m,[24] and P=1.33×10^{-2} Pa, λ_{mean}=3.30 m is obtained. Compared with the 2-mm source-to-substrate distance, this value indicates a ballistic effect during sublimation. The growth rate in a ballistic fashion is believed to be more effective than that of an inert gas flowing in a typical physical vapor transport.[25] Under these circumstances, abundant Bi nanograins nucleated on the top substrate at the early stage of vapor condensation, as shown by the SEM results (Figure S4). This observation agrees with the fact that there is a polycrystalline layer accompanied by rounded and equiaxed rather than lath-like and elongated nanograins, as the substrate temperature is much higher than the melting point of the starting material,[26] as shown by the cross-sectional TEM image (Figure S5). Bi grains with a larger size (submicrons, 100–200 nm in diameter) than the nanograins (below 100 nm, labeled as red arrows in Figure S5) were

occasionally observed, which could also be rationalized by thermal-induced coalescence by consuming adjoining smaller nanograins that would otherwise be equal in size. Figure S5 provides further evidence that the development of two adjacent nanograins is often associated with substantial amounts of large dihedral angles. The dominant large dihedral angle is more favorable because of its lower surface energy, ascribed to a synergistic result from the reduced source-to-substrate distance and the high growth temperature.

A finer examination of the SEM image also shows a radial layer-by-layer growth commensurate screw dislocation, presumably owing to the VS mechanism (Figure S6),[27] as found for typical UV-luminescent ZnO NW.[28] In contrast, the high-temperature treatment and the rapid cooling presented in our VS system could introduce considerable compressive stress at the Bi-film/SiO_2/Si-substrate interface. That is, in our samples, the difference of the thermal expansion coefficient, α', between the Bi film and the SiO_2/Si-substrate is mainly responsible for the presence of this high compressive stress (α'_{Bi}=1.34×10^{-5} K^{-1}; $\alpha'_{\mathrm{SiO_2}}$=0.5×10^{-6} K^{-1}; α'_{Si} =2.4×10^{-6} K^{-1}),[14] which thereby leads to the high-yield Bi NWs. In this connection, Bi NWs appeared to grow from the Bi film surface, being extruded across the grain boundary (labeled as white arrows, Figure S5) into a well-crystallized form approximately 6 μm in length, analogous to the cases of Ni, Sn, and Zn undergoing the thermally-induced stress.[29–31]

Alternatively, because the critical nuclei diameter, D', is inversely proportional to temperature, the sizes of the relative abundances of the Bi nanograins produced on the Si substrate decrease as the growth temperature increases, as predicted by the following equation:[32]

$$D' = \frac{4\sigma\Omega}{RT'\ln(P_1/P_2)}, \qquad \text{(S10)}$$

where σ, Ω, R, T', P_1, and P_2 are the interfacial energy, the molar volume, the ideal gas constant, the temperature of the substrate, the partial pressure of the growth materials, and the pressure of the growth materials at equilibrium, respectively. This trend is similar to that observed in the VS-fabricated analogs,[33] indicating that polycrystalline Bi films continually develop specific surfaces, along with the clustering of increasingly oriented nanograins, hence, plenteous grain boundaries will appear, which accounts for the high-yield NWs in a close-packed manner.

Rather than attempting to harvest NWs via thermal evaporation under high vacuum, normally ranging from 10^{-8} to 10^{-6} torr,[2–4,7,8] we also demonstrate how, by tailoring the reduction reaction, a Bi NW with good crystallinity can be directly derived from a low vacuum. Considering that the growth process was implemented at a rather low pressure of 10^{-4} torr, we

suggest that the formation of Bi oxides or suboxides could be triggered by including of small amounts of oxygen. To prevent this, a hollow graphite cylinder, as a spacer between the top substrate and the bottom starting materials, was adopted in our VS system, making its use for the reduction reaction viable; that is,

$$C+1/2\,O_2 \rightarrow CO \quad (S11)$$

$$2Bi_2O_3+3C \rightarrow 4Bi+3CO_2 \quad (S12)$$

$$Bi_2O_3+3CO \rightarrow 2Bi+3CO_2 \quad (S13)$$

Accordingly, the carbothermal reduction process at 800–900 °C that involves the reaction of gaseous CO and CO_2 is expected to suppress the occurrence of both Bi oxides and suboxides effectively and, consequently, give rise to more intensive growth of Bi NWs.

Section S9, Metallic absorption loss and plasmonic strength in Bi core:
Given that the metallic absorption loss is intrinsically on the order of 10^6 m^{-1} (*Sections S7*, Supporting Information), the α_{loss}^{P} of Bi at λ_P is unignorably large and dominates the overall net gain even for a micrometer-sized short cavity length. In addition, we assumed that η_{in}=1 in a rate-equation analysis owing to the large α_0 of β-Bi_2O_3 semiconductors up to 2×10^6 m^{-1},[34] as found for typical emissive ZnO, GaN, and perovskite analogs.[35–37] As shown in Equations (S1), (S3), and (S6), both α_{loss}^{P} and α_0 predominantly damped the excitation and, consequently, the absence of a population inversion in N_2. It seems impossible to lase because of its high material loss in Bi. However, this result is somewhat contradictory to implying an alternative mechanism of intracavity net gain.

As pioneered by Einstein in 1917, the transitions were modeled by a two-level system through the *A* and *B* coefficients, from which σ_e is of the same order as σ_a, as represented by luminescent semiconductors, oxides, and organics.[38–40] However, when σ_e is increased to two orders of magnitude higher than σ_a (i.e., 10^{-11} vs. 10^{-13}, Table S4), stimulated emission turn-on surge occurs when the overall net gain is high enough to compensate for the total losses. In contrast, the laser output ceases once σ_e is reduced to its original 10^{-13}-m^2 order. Indeed, it can be understood from the corresponding buildup in the photon lifetime (Equation S2), where a σ_e of 1.00×10^{-11} m^2, steady-state N_2 of approximately 2.59×10^{16} #/m^3, and α_{loss}^{L} of 5.14×10^6 m^{-1} are predicted for the laser output when t_c>0, but not vice versa for a decreased σ_e down to 1.00×10^{-13} m^2. This observation can be phenomenologically rationalized by the optical-gain spectrum shown in Figure S19 (cf. *Section S10*, Supporting Information). The above result also implies an intracavity PL strength of up to ca. 100 fold due to the overlapping SPR band

and PL spectrum of the gain medium, similar to those observed in Au- and Ag-based core–shell nanorods (PL strength: 10–100 fold)[41–44] and related plasmonic nanostructures (PL strength: 100–500 fold).[45–50]

Section S10, Optical-gain spectrum:

To better understand mode selection across a broad wavelength span, we performed a systematic optical-gain distribution for σ_a and σ_e. According to the time-dependent rate equations and the resultant parameters, the optical-gain spectra, $G(\lambda)$, upon various levels of excitation in our devices can be described by the following relation:[38]

$$G(\lambda) = N_T[p\sigma_e(\lambda) - (1-p)\sigma_a(\lambda)], \tag{S14}$$

where p is the population fraction of the N_2. Considering all the data in Table S4, the expected $G(\lambda)$ as a function of p is presented in Figure S19. N_2 was unpopulated for p=0; all populations were in N_2 for p=1. As shown in Figure S19a, having a σ_e value of only 1.00×10^{-13} m^2, no effective $G(\lambda)$ exists for all wavelengths, given the high $\alpha_{loss}(\lambda)$ denoted by the gray line. In contrast, Figure S19b shows that the significantly effective $G(\lambda)$ was readily activated by the 100-fold enhanced σ_e of 1.00×10^{-11} m^2 at 350 nm, as addressed earlier.

Section S11, Emission cross section, effective cavity reflectivity, and effective mode radius:

For lasing to occur, in which the round-trip gain compensates for the total cavity loss, the laser threshold condition $N_2\sigma_e \geq N_1\sigma_a + 2\pi n/\lambda_L Q$ is valid, where Q is the quality factor of the laser mode, and the total carrier number N_T is N_1+N_2. The contribution of the overall loss was included in the experimentally determined Q factor. In this connection, it becomes obvious that lasing can be achieved in the F–P cavity that satisfies the minimum fraction of steady-state excited carriers:

$$\frac{N_2}{N_T} = \frac{\sigma_a + 2\pi n/N_T\lambda_L Q}{\sigma_a + \sigma_e}. \tag{S15}$$

According to the laser simulation (Figure 4), lasing turns on at any I_P above the lasing threshold of 12 kW/cm^2 where the clamped N_2 and the N_T were approximately 2.59×10^{16} and approximately 1.53×10^{19} #/m^3, respectively, with a Q factor of approximately 500 and a σ_a of 0.59×10^{-13} m^2, σ_e is calculated as approximately 3.65×10^{-11} m^2. This value matches well with the numerical prediction of 1.00×10^{-11} m^2.

Further, one can reasonably estimate the effective cavity reflectivity R in terms of Q using the following equation[51]

$$Q = \frac{2\pi nd}{\lambda_L}\left[\frac{\sqrt{R\mathrm{e}^{-2\alpha_{\mathrm{loss}}^{\mathrm{L}}d}}}{1-R\mathrm{e}^{-2\alpha_{\mathrm{loss}}^{\mathrm{L}}d}}\right]. \tag{S16}$$

From all the data in Table S4, we found R~75.9%. Despite the low reflectivities in R_1 and R_2 implying no laser action, an increase in R indicates that it would be as effective as a 100-fold strength in σ_e. It is mainly responsible for the loss compensation, as mentioned.

To further verify the effectiveness of our numerical model, we compute the effective mode radius r at λ_L as[52]

$$r = \left[\frac{\pi d\lambda_L\sqrt{R}}{8n(1-R)}\right]^{1/2}. \tag{S17}$$

Using the parameters for the R=75.9%, the effective r is derived as approximately 1.55 μm, close to the numerically determined value of 0.84 μm (Table S4).

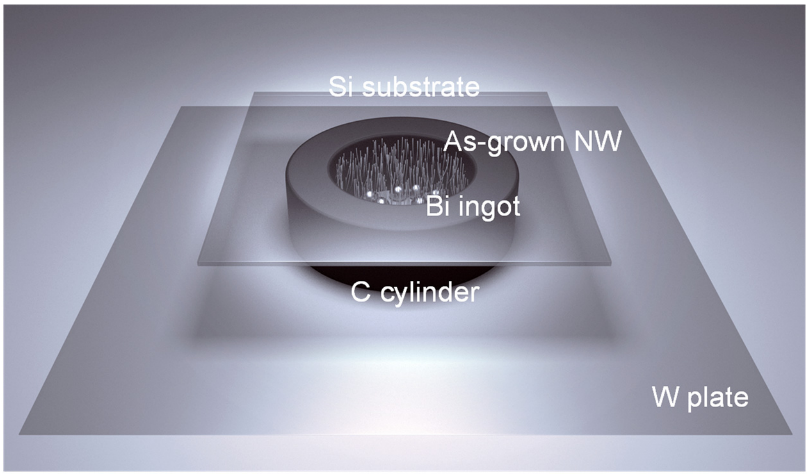

Figure S1. Schematic fabrication of core–shell metal–semiconductor heterojunction NWs with a home-built VS system. The process comprises four steps: source heating and sublimation, nucleation, and growth of target materials via thermal-induced stress.

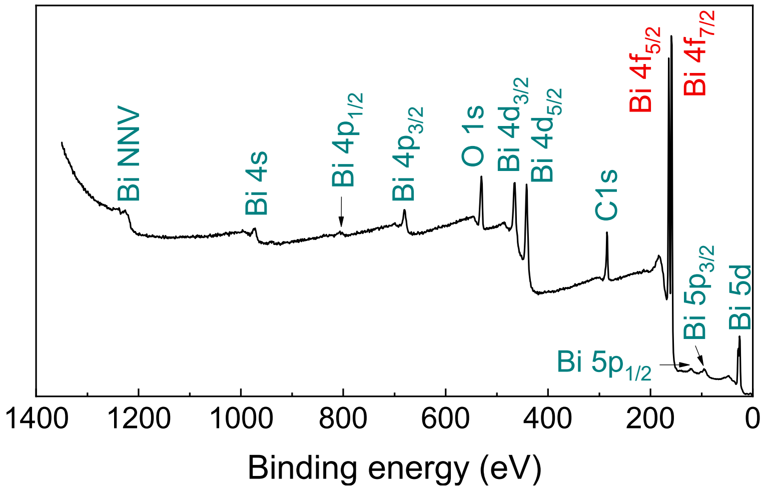

Figure S2. XPS survey spectrum of core–shell metal-semiconductor heterojunction NWs. The wide-scan result shows no signals of elements other than C, Bi, and O, indicating the effectiveness of the proposed VS technique and the purity of as-prepared heterojunction samples.

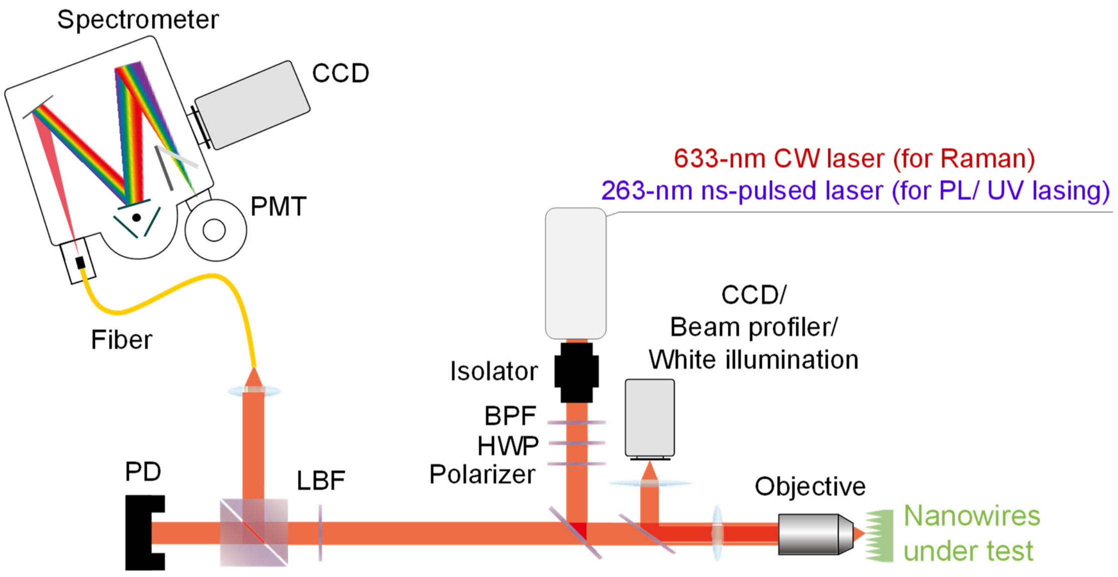

Figure S3. Schematic illustration of home-built high-spatial resolution confocal micro-Raman, micro-PL, and UV lasing experimental setups. Optical characterizations were conducted using a home-built laser confocal microscope, capable of performing position-dependent spectroscopic investigations. Two excitations, i.e., 633 and 263 nm, were focused onto the sample by the objective lens in an upright configuration. All the optical experiments were performed at RT. (BPF: band-pass filter; HWP: half-wave plate; LBF: laser blocking filter; PMT: photomultiplier tube; CCD: charge-coupled device; PD: photodetector)

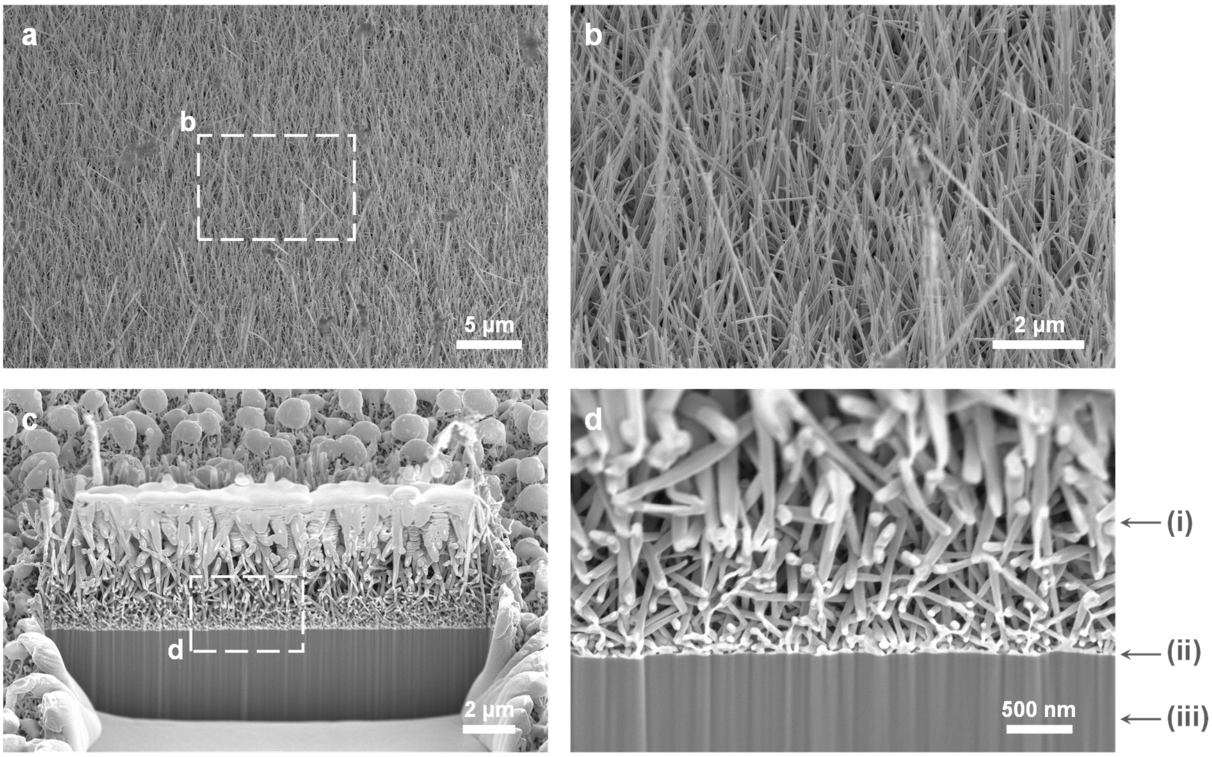

Figure S4. SEM investigations of the core–shell metal–semiconductor heterojunction NWs directly grown on Si substrate. a) Low magnification, showing an oriented and dense distribution. b) High magnification from the square region of (a). c) Cross-section image taken in (b) and cut by FIB milling. A thin layer of amorphous C was deposited on the sample to avoid electrostatic charging before FIB milling. d) Close view of the square region marked in (c), revealing (ii) a thin Bi_2O_3/Bi layer sandwiched between (i) the dense NWs and (iii) Si substrate, as labeled by arrows. All the images were taken at a tilt angle of 52°.

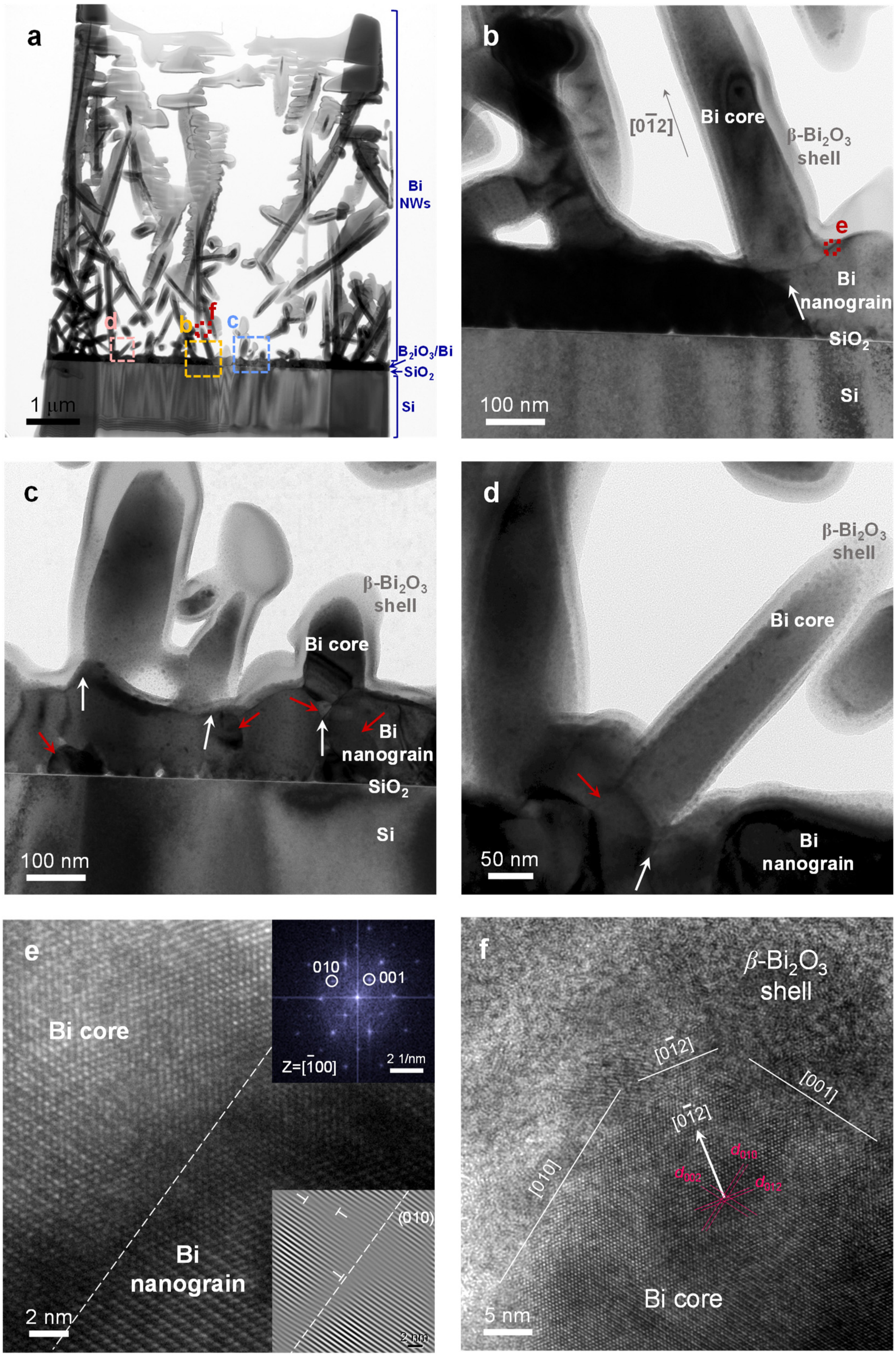

Figure S5. TEM investigations of the core–shell metal–semiconductor heterojunction NWs directly grown on Si substrate. a) Low-magnified image at the NW/Si interface with selected regions for detailed analyses showing an in-between polycrystalline Bi_2O_3/Bi layer beneath the dense NWs, comprising some tiny nanograins (<100 nm in diameter, clearly visible

and marked by red arrows in (b–d)). b–d) TEM images of several of the oriented core–shell metal–semiconductor heterojunction NWs subjected to thermal-induced stress where the NW growth is along <012>, as detailed in (e,f). The NWs initiated out of the nanograin boundaries (marked by white arrows) rather than on the surfaces of nanograins. e) High-resolution lattice image of enlargement in (b) showing the NW impinged over the {010} vicinal plane to form a coherent yet slightly distorted boundary (delineated by dashed lines), as confirmed by the 2-D forward Fourier transformation (upper inset) and the inverse Fourier transform (lower inset) with edge dislocations having a half plane parallel to {001}(denoted by T) as viewed edge-on in the $[\bar{1}00]$ zone axis. f) High-resolution lattice image of one of NW apexes with well-developed {001} and $[0\bar{1}2]$ facets.

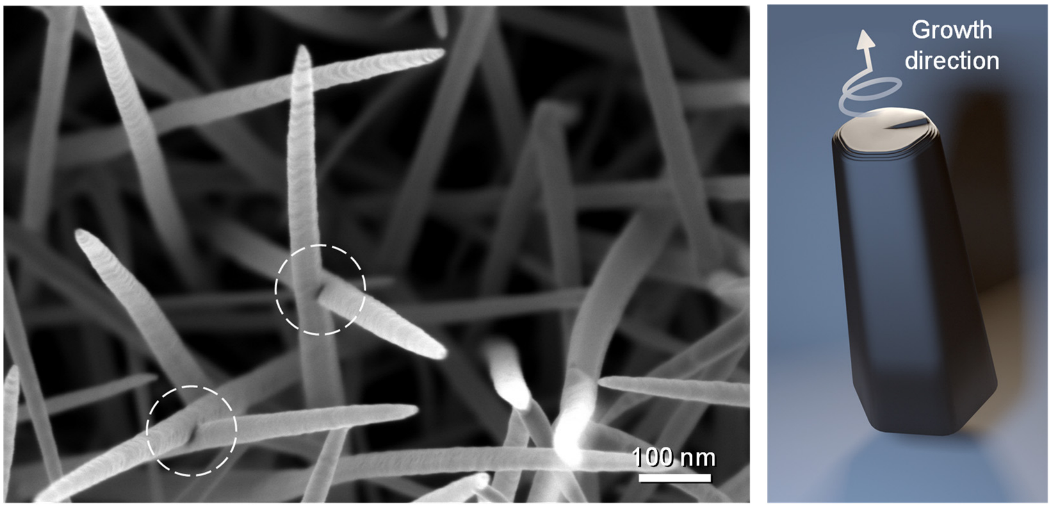

Figure S6. Detailed Morphology of the as-grown core–shell metal–semiconductor heterojunction NWs. The circles highlight cross-overs, clearly indicating the NWs did not grow simultaneouly;[53] hence, the length differences among NWs, as evidenced in Figure 1c. Another salient feature is the onset of NW growth given a radial layer-by-layer structure in both edges of the NW where screw dislocation may participate during the growth, illustrated on the right-hand side.

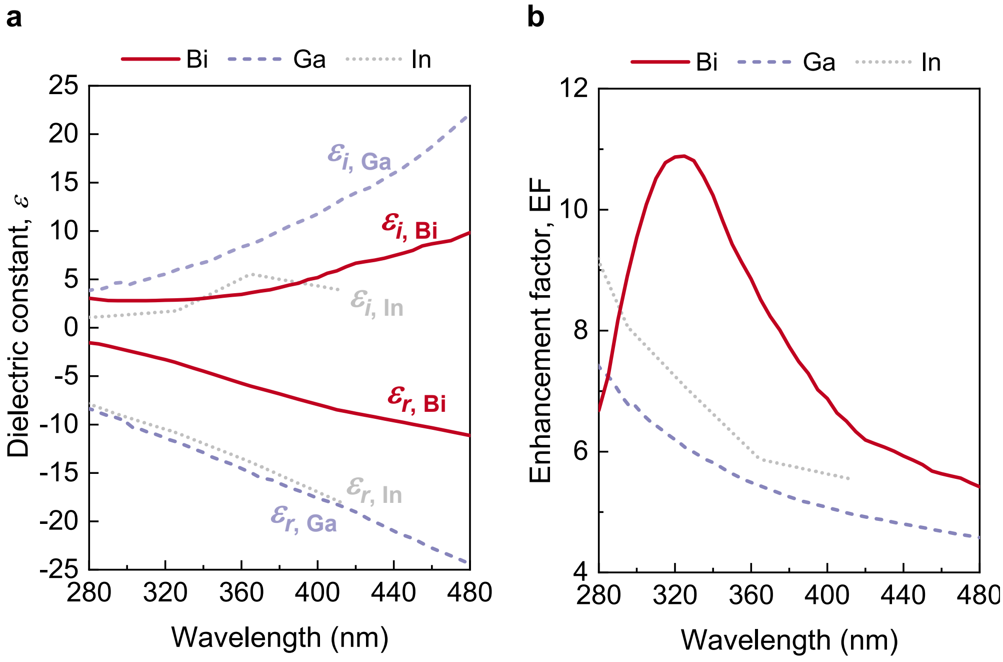

Figure S7. Estimation of the plasmonic enhancement in UV range. a) UV dielectric functions of Bi, Ga, and In, respectively, as labeled in the inset. b) The corresponding enhancement factors of (a) features the effectiveness of plasmonic enhancement of Bi than toxic and costly metals of Ga and In in the 300–400 nm range.

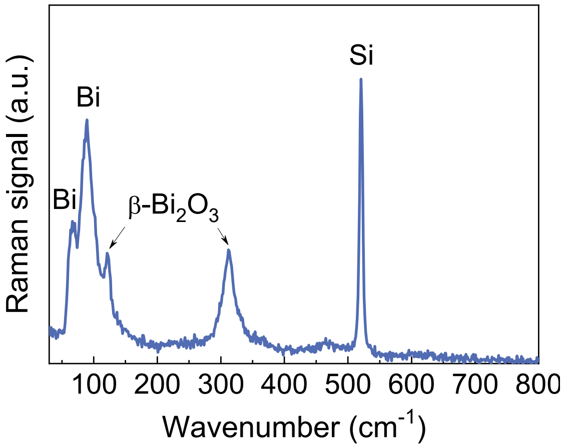

Figure S8. Raman spectrum of the pure β-Bi_2O_3 NWs by intentionally thermal-induced oxidation. For the frequency below 120 cm^{-1}, two pronounced Raman signals come from vibration modes of heavy Bi atoms, attributed to the original Bi nanograins underneath the β-Bi_2O_3 NWs; while above 120 cm^{-1}, two bands are governed by the Bi–O vibrations in β-Bi_2O_3.[54]

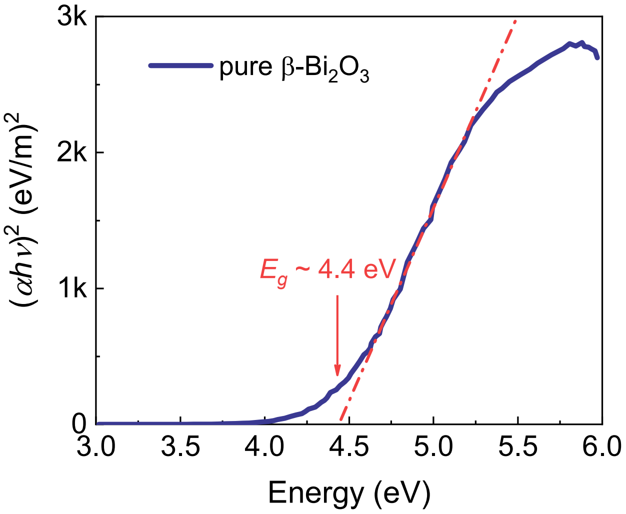

Figure S9. Estimation of the bandgap of the pure β-Bi_2O_3 NW gain medium from extinction spectrum. By the extrapolation of linear approximation, the obtained bandgap is ca. 4.4 eV, which is consistent with the measured micro-PL at 350 nm.

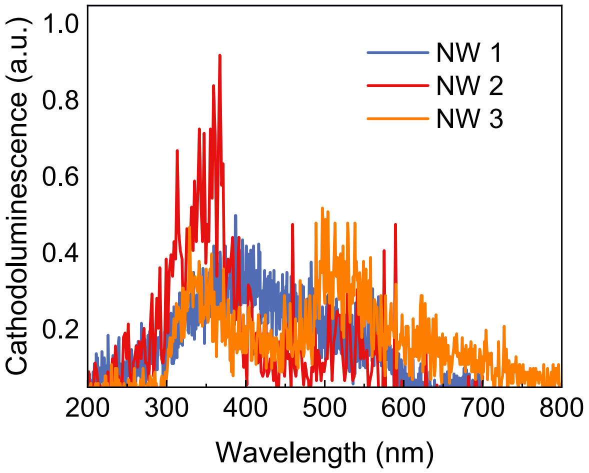

Figure S10. Exemplary CL spectra from the test sample. CL spectra of different single heterojunction NW, which are indicative of the PL spectrum of an ensemble of heterojunction NWs.

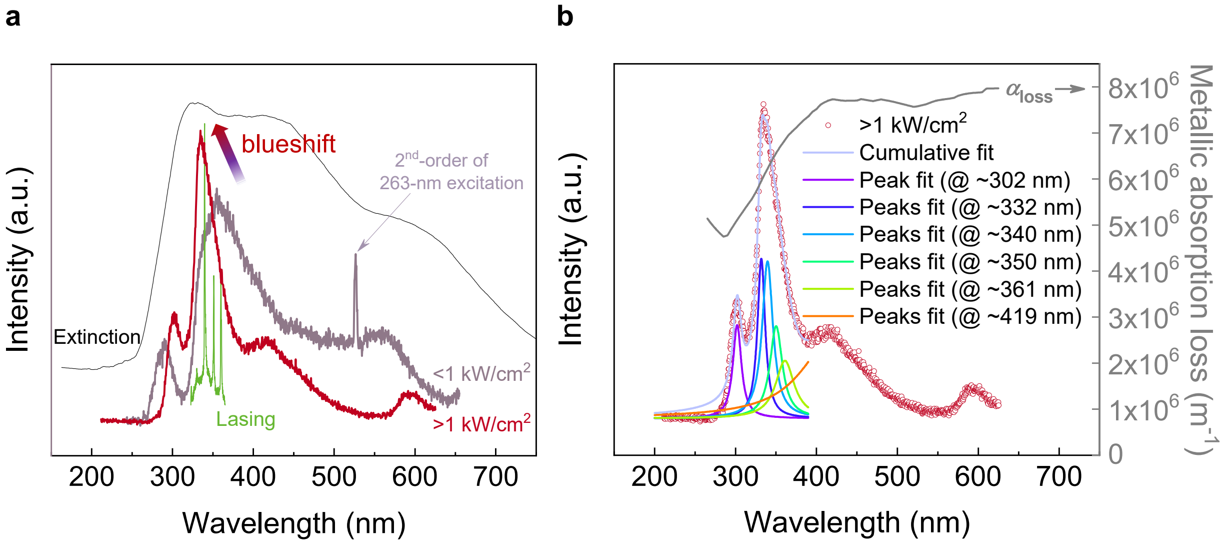

Figure S11. Enhanced optical-gain spectrum via band filling below lasing threshold. a) At a power density higher than 1 kW/cm^2, the blueshift optical-gain peak (red) were observed and enhanced with prominent spectral narrowing than that lower than 1 kW/cm^2 (gray). The band filling promotes a good overlap between the optical-gain spectrum (red) and the extinction spectrum (black), thus benefiting the laser action (green). b) Optical-gain spectrum in (a) for four well-pronounced cavity modes at 330–360 nm, which coincide with the cavity resonance. Spectral widths at resonant modes of 332, 340, 350, and 361 are approximately 11, 16, 18, and 28 nm, respectively. Linewidth variation relative to the ideal F–P resonance is ascribed to energy losses due to parasitic metallic absorption (α_{loss}).

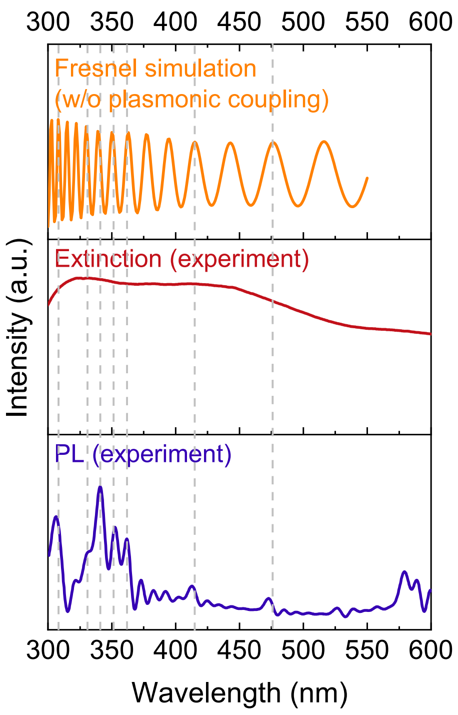

Figure S12. Spectral responses among the F–P interference, the extinction, and the PL. Top panel: Plot of spectral response (calculated by Equation (2), main text) showing a broad modulation due to multiple interferences at each interface, as expected from the classical Fresnel relation. Center panel: Extinction spectrum of pure β-Bi_2O_3 shell. Bottom panel: Polarization-dependent PL spectrum, showing several cavity modes with the F–P characteristics agreeing well with the calculated Fresnel spectrum in the top panel, as marked by dashed lines.

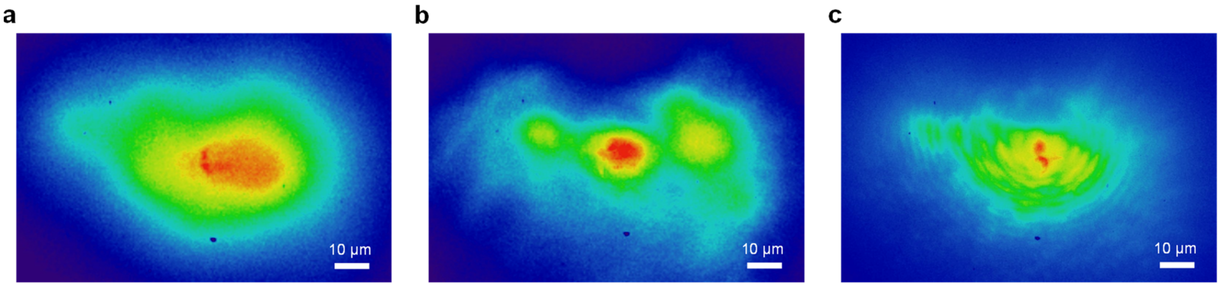

Figure S13. Emission images captured by using a camera-based beam profiler. Images (a,b) below (~4 and ~8 kW/cm^2) and (c) above (~18 kW/cm^2) the laser threshold, where laser action is indicated by distinct interference fringes.

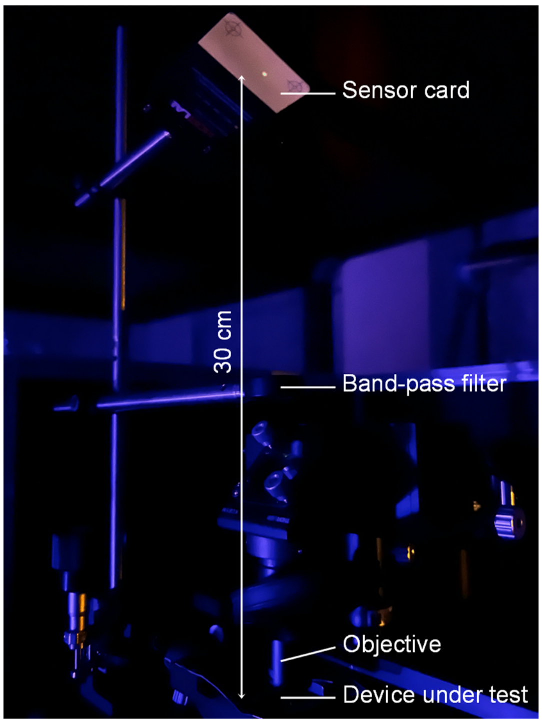

Figure S14. Photograph of directional lasing. Far-field pattern of directional laser output showing high coherent emission above the laser threshold.

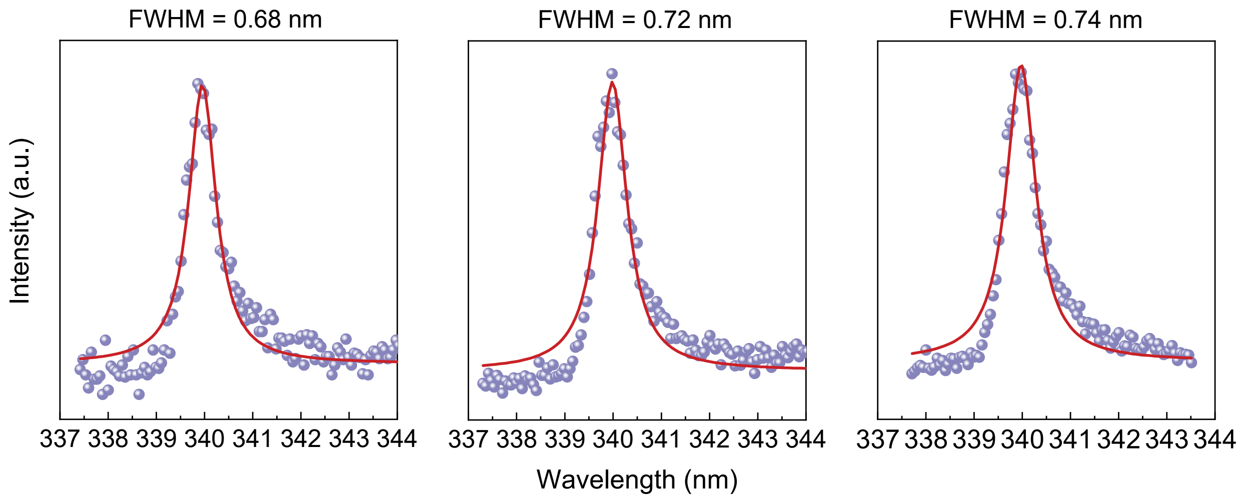

Figure S15. FWHM of the lasing peak from different NW samples. Red curves represent the Gaussian fits to the lasing peaks, showing FWHMs below 0.8 nm and the *Q* factors of ca. 500.

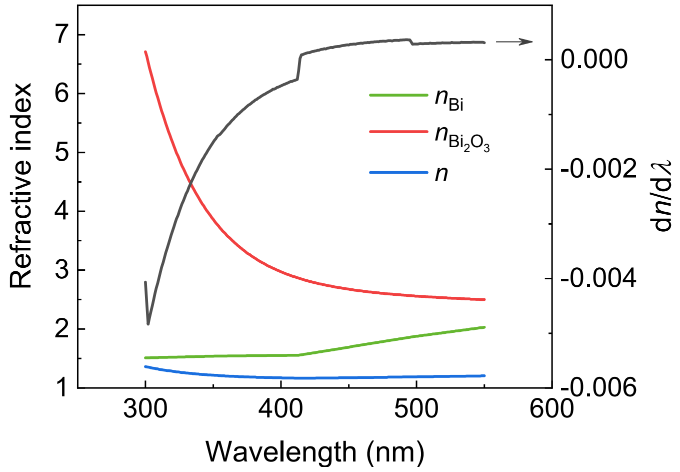

Figure S16. Dispersion relation employed in the numerical calculation. The effective refractive index n is given by $n = [Fn_{\mathrm{Bi}} + (1-F)n_{\mathrm{Bi_2O_3}}] \times F' + n_{\mathrm{air}} \times (1\text{-}F')$ through the core-to-shell volume ratio ($F \approx 70\%$), the average filling factor ($F' \approx 17.5\%$), and the refractive indices of Bi (n_{Bi})[18] and Bi2O3 ($n_{\mathrm{Bi_2O_3}}$)[19], respectively.

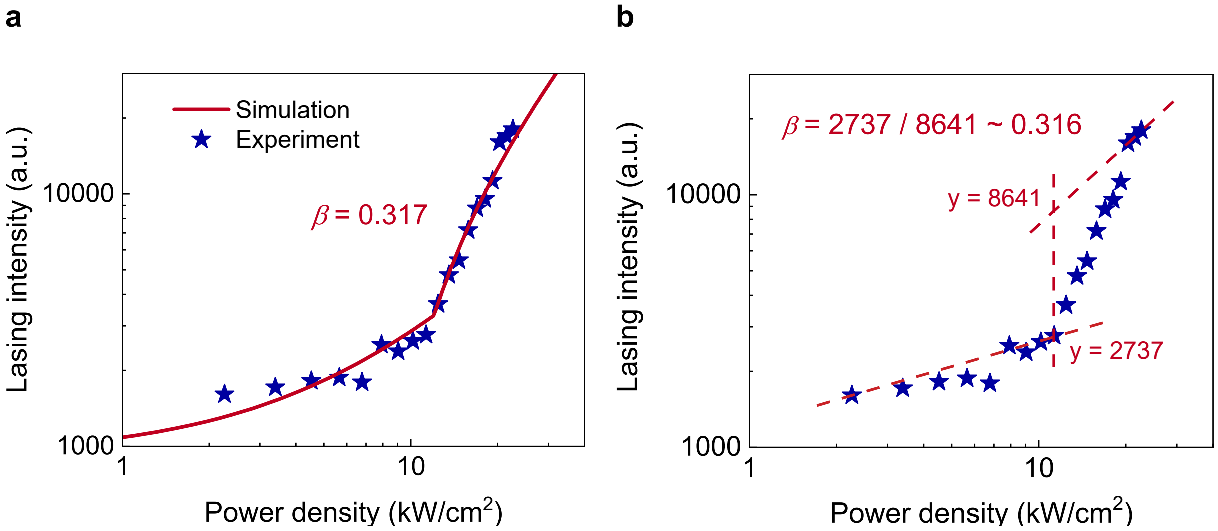

Figure S17. Light-in to light-out curve for the core–shell metal–semiconductor heterojunction NW laser showing its high-*β* factor at low pump power densities. a) A lasing simulation with best-fit spontaneous emission coupling factor *β* of 0.317 is delineated as a solid line, in agreement with the measurements. The data points are the same as those in Figure 3d. b) The upper and lower dashed lines at the threshold kink exhibit output intensities of 8641 and 2737, respectively. The *β* value then turned out to be approximately 0.316 (=2737/8641),[20] which is very close to that in (a).

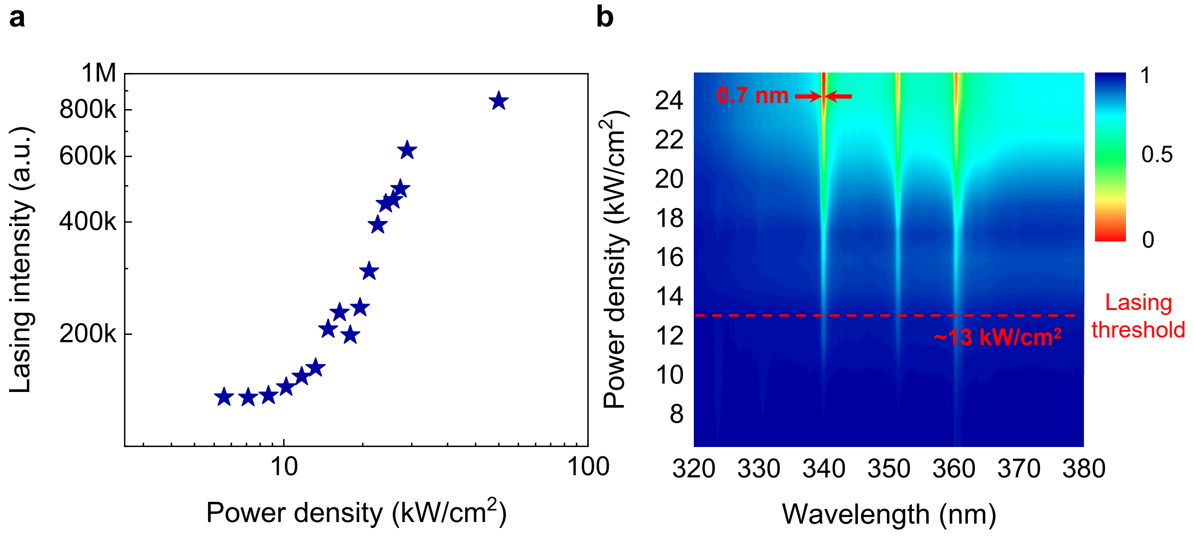

Figure S18. Laser performance for the same NW device tested after 1 year. a) Light-in to light-out curve having an identical lasing threshold of approximately 13 kW/cm^2, demonstrating the effectiveness of device reliability for future mass production. b) The corresponding spectral evolution of (a) recorded at RT.

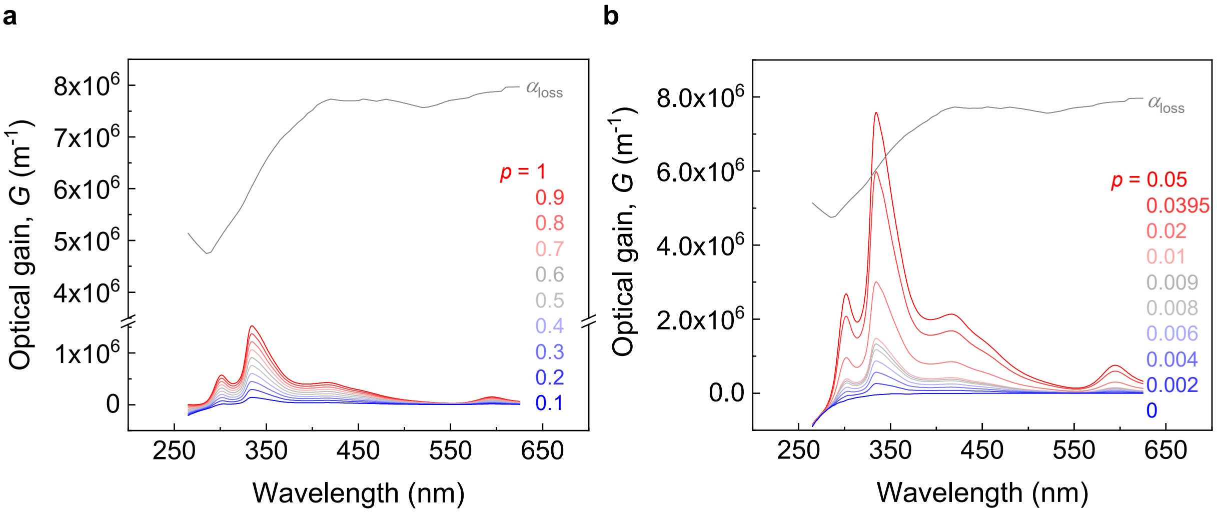

Figure S19. Plasmonic-enhanced optical-gain spectrum in core–shell metal–semiconductor heterojunction NWs. Optical-gain spectra based on Equation (S14) shown alongside parasitic metallic loss for the σ_e and σ_a of a) 1.00×10^{-13} and 0.59×10^{-13} m^2; b) 1.00×10^{-11} and 0.59×10^{-13} m^2, underscore the role that the plasmonic effect plays in realizing the low-threshold and RT UV laser action.

Table S1. Summary of the performance of the reported ZnO lasers. (in descending order of threshold)

Morphology	Fabrication	Resonant mode	Threshold (kW/cm^2)	Temperature (K)	Excitation (nm)	Lasing (nm)	Pulse width	FWHM (nm)	References
Microsphere	Laser ablation	Plasmonics	-	RT	325	389.6	100 fs	0.68	[55]
NW	Vapor-liquid-solid (VLS)	F–P	-	RT	310	387	100-200 fs	0.25	[56]
NW	VLS	F–P	-	RT	349	387	10 ns	-	[57]
Microneedle	Vapor phase transport (VPT)	F–P	10^9	RT	800	395-405	150 fs	0.37	[58]
NW	Chemical vapor deposition (CVD)	F–P	0.7×10^9	RT	798	392	150 fs	0.5	[59]
NW	VPT	F–P	25.4×10^6<br>31.9×10^6	RT	325	386 – 390<br>378, 382	150 fs	<0.5<br><1.5	[60]
NW	VPT	F–P	17.3×10^6	10	355	369	150 fs	0.1	[61]
NW	VPT	F–P	2×10^6	RT	700	390	80 fs	0.2	[62]
NW	VLS	Plasmonics	1.89×10^6	RT	355	370	150 fs	-	[63]
Nanosphere	Hydrothermal	Excitonics	1.89×10^6	RT	280	390	100 fs		[64]
NW	CVD	F–P	1.82×10^6	RT	355	385-390	1.1 ns	0.6	[65]
Nanoribbon	VLS	F–P	1.69×10^6	RT	310	~390	200 fs	0.1-0.5	[66]
NW	-	Plasmonics	1.33×10^6	RT	355	375	150 fs	-	[67]
Microrod<br>Nanobelt	VPT	WGM<br>F–P	0.5×10^6<br>0.67×10^6	RT	325	390	150 fs	0.4<br>0.42	[68]
NW	Hydrothermal	Plasmonics	80×10^3	77	355	370	0.5 ns	<1	[69]
NW	Hydrothermal	F–P	60×10^3<br>10^6	77<br>RT	355	373	0.5 ns	0.4	[70]
NW	Hydrothermal	Plasmonics	52×10^3	77	355	372	0.5 ns	<0.5	[71]
NW	Drop-casting	F–P	20×10^3	RT	355	377<br>381	1 ns	<2	[72]
NW	Hydrothermal	F–P	16×10^3<br>110×10^3	77<br>RT	355	372<br>381	0.5 ns	0.2	[73]
NW	Hydrothermal	F–P	11×10^3	77	355	373	0.5 ns	0.5	[74]
NW	Hydrothermal	Plasmonics	12.2×10^3<br>1.68×10^3<br>0.56×10^3	RT<br>273<br>77	355	381<br>375<br>370	0.5 ns	0.3	[75]
NW	VPT	F–P	6270	RT	355	378	-	1	[76]

Table S1. Summary of the performance of the reported ZnO lasers. (in descending order of threshold)

Morphology	Fabrication	Resonant mode	Threshold (kW/cm^2)	Temperature (K)	Excitation (nm)	Lasing (nm)	Pulse width	FWHM (nm)	References
NW	-	Plasmonics	3500<br>30×10^3	RT<br>360	266	383<br>387	0.35 ns	0.2	[77]
NW	VPT	F–P	2000-5000	RT	349	387.5	10 ns	-	[78]
NW	VPT	WGM	800	RT	325	392.7	150 fs	0.08	[79]
NW	VPT	Random	420<br>300~480	RT	355	379<br>383	0.120 ns	0.4	[80]
NW	Thermal vapor	WGM	170	10	266	~372	10 ns	0.1	[81]
NW	Hydrothermal	DBR	160	77	355	373	0.5 ns	0.22	[82]
NW	VPT	F–P	150	RT	285	380.8<br>384	< 1 ps	1.2	[83]
NW	CVD	F–P	135	RT	355	390-396	100 fs	0.22	[84]
Microbelt	VPT	F–P	120	RT	325	390	-	-	[85]
NW	CVD	WGM	100<br>162	RT	355	390-396	100 fs	0.11	[86]
NW	CVD	WGM	88	RT	355	392-396	100 fs	0.06	[87]
NW	Chemical vapor transport (CVT)	F–P	50	RT	266	387-395	8 ns	0.8	[88]
Nanorod	Low pressure metal organic vapor phase epitaxy (MOVPE)	Random	40	RT	355	390	6 ns	0.4	[89]
NW	VLS	F–P	40	RT	285	385	<300 fs	0.3	[90]
NW	VPT	F–P	40	RT	266	383	3 ns	0.3	[91]
Nanotube	MOVPE	F–P	50<br>35	RT	355	390<br>390	6 ns	1.7<br>0.9	[92]
Nanodisk	CVT	WGM	62.5<br>31.25	RT<br>8	266	391<br>375	8 ns	0.8	[93]
Thin film	Pulsed laser deposition	F–P	15	RT	343	378	250 fs	-	[94]
**NW**	**Modified vapor-solid deposition**	**Coupled F–P/plasmonics**	**12**	**RT**	**263**	**340-360**	**1 ns**	**0.7**	**Our work**

Table S2. Summary of the performance of the reported group III–nitride semiconductors lasers. (in descending order of threshold)

Gain material	Morphology	Fabrication	Resonant mode	Threshold (kW/cm^2)	Temperature (K)	Excitation (nm)	Lasing (nm)	Pulse width	FWHM (nm)	References
2,4,5-triphenylimi-dazole	NW	Adsorbent-assisted physical vapor deposition	F–P	-	RT	325	375	200 fs	~3	[95]
AlGaN	Thin film	Expitaxial Growth	DBR	10^4	RT	266	310	1.3 ns	0.15	[96]
$AlN/Al_{0.35}Ga_{0.65}N$	Microdisk	Metal-organic chemical vapor deposition (MOCVD)	WGM	2420	RT	193	300.1	10 ns	1	[97]
AlGaInN	Thin film	MOCVD	F–P	800	RT	308	340	20 ns	-	[98]
AlGaN	Thin film	Plasma-assisted molecular beam epitaxy (MBE)	F–P	800	RT	266	303	-	-	[99]
$Al_xGa_{1-x}N/Al_yGa_{1-y}N$		Low pressure MOCVD	DBR	520	RT	266	343.7	1 ns	2	[100]
ZnS	Nanosheet	Tube furnace	Random	200	RT	266	332	0.12 ns	0.4	[101]
ZnS	NW	Tube furnace	F–P	130	RT	266	338	5 ns	0.3	[102]
**Bi/Bi_2O_3 core–shell**	**NW**	**Modified vapor-solid deposition**	**Coupled F–P/plasmonics**	**12**	**RT**	**263**	**340-360**	**4.41 ns**	**~0.7**	**Our work**

Table S3. Summary of the performance of the reported GaN lasers. (in descending order of threshold)

Gain material	Morphology	Fabrication	Resonant mode	Threshold (kW/cm^2)	Temperature (K)	Excitation (nm)	Lasing (nm)	Pulse width	FWHM (nm)	References
GaN	NW	Nckel-catalyzed chemical vapor transport	F–P	-	RT	266	370	8 ns	0.6	[103]
GaN	Microdisk	Dry and wet etching	WGM	-	4.3	325	~365	150 fs	-	[104]
GaN	Membrane	MOCVD	F–P	7.56×10^6 10.11×10^6 11.2×10^6	RT	325	370	150 fs	-	[105]
GaN	NW	Low pressure MOCVD	F–P	1.4×10^5	RT	355	363	0.5 ns	0.3	[106]
GaN	Nano gammadion coated Al	MOCVD	F–P	29.8×10^3 37.6×10^3	RT	355	364	0.5 ns	0.35	[107]
InGaN/GaN	2D Photonic quasicrystal	Low pressure MOCVD, Nanoimprint lithography	DFB	18×10^3 48×10^3	RT	355	366 366	0.5 ns	- 1.5	[108]
GaN	Nanorod cladded Al	MOCVD	WGM	10.4×10^3	RT	355	362.5 364.3 365.2	0.5 ns	-	[109]
GaN/AlGaN	NW	CVT	F–P	$(20\pm10)\times10^3$	RT	310	384	100-200 fs	-	[110]
GaN	NW	Ammonia-based MBE+ Electron-beam lithography	F–P	8149	RT	266	369	0.35 ns	0.26	[111]
GaN	NW	MOCVD	F–P	3500	RT	355	369	Nanosecond	0.8	[112]
InGaN/GaN	NW	Ammonia-based MBE+ Electron-beam lithography	F–P	3000	RT	266	370	0.35 ns	0.1	[113]
GaN	NW	MOCVD, etching	F–P	2400 4900	RT RT	266	364 366	0.4 ns	0.23 0.18	[114]
GaN	NW	MOCVD	Random	1800-3000 3400-5600	RT	266	373	3-5 ns	<1	[115]
GaN	NW	MOCVD+EBL+reactive ion etching	Micostadium	1536	RT	266	372	7 ns	0.9	[116]
GaN	Micro-chimney	Photolithography+ inductive coupled plasma etching	WGM	1050	RT	355	371.96	6 ns	0.17	[117]
GaN	NW	MOCVD	F–P	874	RT	266	370	0.4 ns	0.14	[118]

Table S3. Summary of the performance of the reported GaN lasers. (in descending order of threshold)

Gain material	Morphology	Fabrication	Resonant mode	Threshold (kW/cm^2)	Temperature (K)	Excitation (nm)	Lasing (nm)	Pulse width	FWHM (nm)	References
GaN	Hexagonal nanoring	Radio-frequency plasma MBE	WGM	750	RT	355	365.6	5 ns	0.3	[119]
GaN	Nanopillar	MOCVD	WGM	420	RT	349	373	4 ns	2.5	[120]
GaN	NW	Tube furnace CVD	F–P	410	RT	355	374	1 ns	0.8	[121]
GaN	Micro-pyramids	MOCVD	WGM	400-500	RT		367.2		0.054	[122]
GaN	Microdisk	Heteroepitaxy	WGM	250	RT	355	370 – 375	6 ns	0.3	[123]
GaN	NW	MOCVD	F–P	241	RT	266	369	0.4 ns	0.12	[124]
GaN	NW	MOCVD,<br>2-step etching	F–-P	231<br>439	RT<br>RT	267	363	100 ps	0.12<br>-	[125]
GaN	Microdisk	Photolithography	WGM	225	RT	355	379.25	6 ns	0.3	[126]
GaN	NW	MOCVD	Random	202.9	RT	266	~375	20 ns		[127]
GaN	NW	MOCVD,<br>2-step etching	DFB	200-400	RT	266	370	0.4 ns		[128]
GaN	NW	Plasma-assisted MBE	F–P	200	RT	350	360	10 ps	1	[129]
GaN/InGaN core–shell	Nanorod array	MOCVD	WGM	140	RT	355	376	-	0.22	[130]
GaN	NW	MOCVD, wet etching	F–P	120	RT	266	375	6 ns	-	[131]
GaN	NW	Plasma-assisted MBE	Photonic Crystal	120	RT	266	371.3	100 fs	0.55	[132]
GaN	NW	MOCVD	F–P	22	RT	266	373	7 ns	0.8	[133]
GaN	NW	Nickel catalyst deposited	F–P	3.5-7	RT	310	370-380	100-200 fs	0.6-1	[134]
GaN	Metal coated nanostripe	MOCVD	F–P	0.042	RT	355	370	0.5 ns	1.8	[135]
GaN,<br>AlN/AlGaN	Microdisk	Low-pressure MOCVD	DBR	0.03<br>0.043	RT	355	377<br>379	0.5 ns	0.9<br>1.2	[136]
GaN	NW	MOCVD	WGM	0.017	RT	355	363.5	-	0.8	[137]
**Bi/Bi_2O_3 core–shell**	**NW**	**Modified vapor-solid deposition**	**Coupled F–P/plasmonics**	**12**	**RT**	**263**	**340-360**	**4.41 ns**	**0.7**	**Our work**

Table S4. Extracted best-fit values for the core–shell metal–semiconductor NW laser.

Parameter	Simulation	Theory	Experiment
Lasing threshold, P_{th} (kW/cm^2)	12		12
Pumping wavelength, λ_p (nm)	263		263
Lasing wavelength, λ_L (nm)	340		340
Pulse width, Δt (ns)	4.41		4.41
Reptition rate (kHz)	1		1
Absorption cross section, σ_a (m^2)	0.59×10^{-13}	1.0×10^{-13} (=α_{abs}/N_T) [34,138]	
Emission cross section, σ_e (m^2)	1.00×10^{-11}	3.65×10^{-11}	
lifetime, τ (ns)	32.8	39.0 [139]	
Absorption loss at λ_P, α_{loss}^{P} (m^{-1})	5.14×10^{6}	8.98×10^{6} [21]	
Absorption loss at λ_L, α_{loss}^{L} (m^{-1})	9.00×10^{6}	9.90×10^{6} [21]	
Spontaneous emission factor, β	0.317	0.316 [20]	
Absorption coefficient at λ_P, α_{abs} (m^{-1})	0.9×10^{6}	2×10^{6} [34]	
Coupling coefficient at λ_P, η_{in}	1		
Coupling coefficient at λ_L, η_{out}	0.9		
Mode radius, r (μm)	0.84	1.55 [52]	
Refractive index at λ_L, n	1.23		1.23
Cavity length, d (μm)	6.1		6.1
Reflectance of input coupler, R_1 (%)	1.03		1.03
Reflectance of output coupler, R_2 (%)	38.48		38.48
Total carrier number, N_T (#/m^3)	1.53×10^{19}	1.93×10^{19} [138]	
Excited carrier number, N_2 (#/m^3)	2.59×10^{16}		

References

[1] C. Xie, X. T. Lu, M. R. Ma, X. W. Tong, Z. X. Zhang, L. Wang, C. Y. Wu, W. H. Yang, L. B. Luo, *Adv. Opt. Mater.* **2019**, *7*, 1901257.

[2] T. Y. Lin, Y. L. Chen, C. F. Chang, G. M. Huang, C. W. Huang, C. Y. Hsieh, Y. C. Lo, K. C. Lu, W. W. Wu, L. J. Chen, *Nano Lett.* **2018**, *18*, 778.

[3] J. Midya, S. K. Das, *Phys. Rev. Lett.* **2017**, *118*, 165701.

[4] Z. Zhang, Y. Wang, H. Li, W. Yuan, X. Zhang, C. Sun, Z. Zhang, *ACS Nano* **2016**, *10*, 763.

[5] S. Wang, Y. He, X. Fang, J. Zou, Y. Wang, H. Huang, P. M. F. J. Costa, M. Song, B. Huang, C. T. Liu, P. K. Liaw, Y. Bando, D. Golberg, *Adv. Mater.* **2009**, *21*, 2387.

[6] Y. L. Chueh, M. W. Lai, J. Q. Liang, L. J. Chou, Z. L. Wang, *Adv. Funct. Mater.* **2006**, *16*, 2243.

[7] J. Zhou, S. Deng, L. Gong, Y. Ding, J. Chen, J. Huang, J. Chen, N. Xu, Z. L. Wang, *J. Phys. Chem. B* **2006**, *110*, 10296.

[8] J. Kim, W. Shim, W. Lee, *J. Mater. Chem. C* **2015**, *3*, 11999.

[9] A. J. Caruana, M. D. Cropper, S. A. Stanley, *Surf. Coat. Technol.* **2015**, *271*, 8.

[10] M. Liu, J. Tao, C. Y. Nam, K. Kisslinger, L. Zhang, D. Su, *Nano Lett.* **2014**, *14*, 5630.

[11] S. A. Stanley, C. Stuttle, A. J. Caruana, M. D. Cropper, A. S. O. Walton, *J. Phys. D: Appl. Phys.* **2012**, *45*, 435304.

[12] J. Ham, W. Shim, D. H. Kim, K. H. Oh, P. W. Voorhees, W. Lee, *Appl. Phys. Lett.* **2011**, *98*, 043102.

[13] S. Lee, J. Ham, K. Jeon, J. S. Noh, W. Lee, *Nanotechnology* **2010**, *21*, 405701.

[14] W. Shim, J. Ham, K. I. Lee, W. Y. Jeung, M. Johnson, W. Lee, *Nano Lett.* **2009**, *9*, 18.

[15] Y. T. Cheng, A. M. Weiner, C. A. Wong, M. P. Balogh, M. J. Lukitsch, *Appl. Phys. Lett.* **2002**, *81*, 3248.

[16] A. Sennaroglu, *Opt. Lett.* **2001**, *26*, 500.

[17] G. M. James, E. M. Harrell, C. Bracikowski, K. Wiesenfeld, R. Roy, *Opt. Lett.* **1990**, *15*, 1141.

[18] J. Toudert, R. Serna, I. Camps, J. Wojcik, P. Mascher, E. Rebollar, T. A. Ezquerra, *J. Phys. Chem. C* **2017**, *121*, 3511.

[19] J. D. T. Kruschwitz, W. T. Pawlewicz, *Appl. Opt.* **1997**, *36*, 2157.

[20] H. Takashima, H. Fujiwara, S. Takeuchi, K. Sasaki, M. Takahashi, *Appl. Phys. Lett.* **2008**, *92*, 071115.

[21] X. Yan, L. Zhu, Y. Zhou, Y. E, L. Wang, X. Xu, *Appl. Opt.* **2015**, *54*, 6732.

[22] K. H. Li, X. Liu, Q. Wang, S. Zhao, Z. Mi, *Nat. Nanotechnol.* **2015**, *10*, 140.

[23] K. A. Jackson, *Kinetic Processes: Crystal Growth, Diffusion, and Phase Transformations in Materials*, Wiley, Hoboken, NJ **2006**.

[24] M. Mantina, A. C. Chamberlin, R. Valero, C. J. Cramer, D. G. Truhlar, *J. Phys. Chem. A* **2009**, *113*, 5806.

[25] C. Kloc, P. G. Simpkins, T. Siegrist, R. A. Laudise, *J. Cryst. Growth* **1997**, *182*, 416.

[26] C. V. Thompson, R. Carel, *J. Mech. Phys. Solids* **1996**, *44*, 657.

[27] S. Cao, C. Guo, Y. Wang, J. Miao, Z. Zhang, Q. Liu, *Solid State Commun.* **2009**, *149*, 87.

[28] S. A. Morin, S. Jin, *Nano Lett.* **2010**, *10*, 3459.

[29] W. Hu, S. Jhulki, W. Fu, L. Chen, F. Wang, K. Turcheniuk, A. Magasinski, G. Yushin, *Chem. Mater.* **2021**, *33*, 5368.

[30] X. Xiao, A. K. Sachdev, D. Haddad, Y. Li, B. W. Sheldon, S. K. Soni, *Appl. Phys. Lett.* **2010**, *97*, 141904.

[31] J. Monk, D. Farkas, *Phys. Rev. B* **2007**, *75*, 045414.

[32] W. A. Tiller, *The Science of Crystallization*, Cambridge University Press, New York, NJ **1991**.

[33] Y. Baek, K. Yong, *J. Phys. Chem. C* **2007**, *111*, 1213.

[34] Q. Y. Li, Z. Y. Zhao, *Phys. Lett. A* **2015**, *379*, 2766.

[35] D. H. Nguyen, J. Y. Sun, C. Y. Lo, J. M. Liu, W. S. Tsai, M. H. Li, S. J. Yang, C. C. Lin, S. D. Tzeng, Y. R. Ma, M. Y. Lin, C. C. Lai, *Adv. Mater.* **2021**, *33*, 2006819.

[36] J. F. Muth, R. M. Kolbas, A. K. Sharma, S. Oktyabrsky, J. Narayan, *J. Appl. Phys.* **1999**, *85*, 7884.

[37] G. Yu, G. Wang, H. Ishikawa, M. Umeno, T. Soga, T. Egawa, J. Watanabe, T. Jimbo, *Appl. Phys. Lett.* **1997**, *70*, 3209.

[38] D. C. Brown, N. S. Tomasello, C. L. Hancock, *Opt. Express* **2021**, *29*, 33818.

[39] Q. Wang, R. Dahal, I. W. Feng, J. Y. Lin, H. X. Jiang, R. Hui, *Appl. Phys. Lett.* **2011**, *99*, 121106.

[40] T. Grossmann, S. Schleede, M. Hauser, M. B. Christiansen, C. Vannahme, C. Eschenbaum, S. Klinkhammer, T. Beck, J. Fuchs, G. U. Nienhaus, U. Lemmer, A. Kristensen, T. Mappes, H. Kalt, *Appl. Phys. Lett.* **2010**, *97*, 063304.

[41] N. S. Abadeer, M. R. Brennan, W. L. Wilson, C. J. Murphy, *ACS Nano* **2014**, *8*, 8392.

[42] S. Y. Liu, L. Huang, J. F. Li, C. Wang, Q. Li, H. X. Xu, H. L. Guo, Z. M. Meng, Z. Shi, Z. Y. Li, *J. Phys. Chem. C* **2013**, *117*, 10636.

[43] T. Ming, L. Zhao, Z. Yang, H. Chen, L. Sun, J. Wang, C. Yan, *Nano Lett.* **2009**, *9*, 3896.

[44] K. Aslan, M. Wu, J. R. Lakowicz, C. D. Geddes, *J. Am. Chem. Soc.* **2007**, *129*, 1524.

[45] C. Liang, J. Luan, Z. Wang, Q. Jiang, R. Gupta, S. S. Cao, K. K. Liu, J. J. Morrissey, E. D. Kharasch, R. R. Naik, S. Singamaneni, *ACS Appl. Mater. Interfaces* **2021**, *13*, 11414.

[46] Z. Eftekhari, A. Ghobadi, M. C. Soydan, D. U. Yildirim, N. Cinel, E. Ozbay, *Opt. Lett.* **2021**, *46*, 1664.

[47] G. Yang, Q. Shen, Y. Niu, H. Wei, B. F. Bai, M. H. Mikkelsen, H. B. Sun, *Laser Photonics Rev.* **2020**, *14*, 1900213.

[48] Y. Luo, X. He, Y. Kim, J. L. Blackburn, S. K. Doorn, H. Htoon, S. Strauf, *Nano Lett.* **2019**, *19*, 9037.

[49] S. I. Bogdanov, M. Y. Shalaginov, A. S. Lagutchev, C. C. Chiang, D. Shah, A. S. Baburin, I. A. Ryzhikov, I. A. Rodionov, A. V. Kildishev, A. Boltasseva, V. M. Shalaev, *Nano Lett.* **2018**, *18*, 4837.

[50] C. Vietz, I. Kaminska, M. S. Paz, P. Tinnefeld, G. P. Acuna, *ACS Nano* **2017**, *11*, 4969.

[51] M. Malak, N. Pavy, F. Marty, Y. A. Peter, A. Q. Liu, T. Bourouina, *Appl. Phys. Lett.* **2011**, *98*, 211113.

[52] T. Enomoto, T. Sasaki, K. Sekiguchi, Y. Okada, K. Ujihara, *J. Appl. Phys.* **1996**, *80*, 6595.

[53] S. A. Stanley, C. Stuttle, A. J. Caruana, M. D. Cropper, A. S. O. Walton, *J. Phys. D.: Appl. Phys.* **2012**, *45*, 435304.

[54] L. Kumari, J. H. Lin, Y. R. Ma, *J. Phys. D: Appl. Phys*. **2008**, *41*, 025405.

[55] X. X. Wang, C. X. Xu, F. F. Qin, Y. J. Liu, A. G. Manohari, D. T. You, W. Liu, F. Chen, Z. L. Shi, Q. N. Cui, *Nanoscale* **2018**, *10*, 17852.

[56] J. C. Johnson, H. Yan, P. Yang, R. J. Saykally, *J. Phys. Chem. B* **2003**, *107*, 8816.

[57] H. Y. Li, S. Rühle, R. Khedoe, A. F. Koenderink, D. Vanmaekelbergh, *Nano Lett.* **2009**, *9*, 3515.

[58] G. P. Zhu, C. X. Xu, J. Zhu, C. G. Lv, Y. P. Cui, *Appl. Phys. Lett*. **2009**, *94*, 051106.

[59] C. F. Zhang, Z. W. Dong, G. J. You, S. X. Qian, H. Deng, *Opt. Lett.* **2006**, *31*, 3345.

[60] J. Lu, M. Jiang, M. Wei, C. Xu, S. Wang, Z. Zhu, F. Qin, Z. Shi, C. Pan, *ACS Photonics* **2017**, *10*, 2419.

[61] H. Zhou, M. Wissinger, J. Fallert, R. Hauschild, F. Stelzl, C. Klingshirn, H. Kalt, *Appl. Phys. Lett.* **2007**, *91*, 181112.

[62] C. Zhang, F. Zhang, T. Xia, N. Kumar, J. I. Hahm, J. Liu, Z. L. Wang, J. Xu, *Opt. Express* **2009**, *17*, 7893.

[63] H. Yu, T. P. Sidiropoulos, W. Liu, C. Ronning, P. K. Petrov, S. H. Oh, S. A. Maier, P. Jin, R. F. Oulton, *Adv. Opt. Mater.* **2017**, *5*, 1600856.

[64] K. Appavoo, X. Liu, V. Menon, M. Y. Sfeir, *Nano Lett.* **2016**, *16*, 2004.

[65] X. P. Huang, Y. L. Liu, P. Wang, K. Chen, Q. Zhao, *Appl. Phys. A* **2015**, *121*, 1203.

[66] H. Yan, J. Johnson, M. Law, R. He, K. Knutsen, J. R. McKinney, J. Pham, R. Saykally, P. Yang, *Adv. Mater.* **2003**, *15*, 1907.

[67] T. P. Sidiropoulos, R. Röder, S. Geburt, O. Hess, S. A. Maier, C. Ronning, R. F. Oulton, *Nat. Phys.* **2014**, *10*, 870.

[68] J. Dai, C. Xu, T. Nakamura, Y. Wang, J. Li, Y. Lin, *Opt. Express* **2014**, *22*, 28831.

[69] Y. C. Chung, P. J. Cheng, Y. H. Chou, B. T. Chou, K. B. Hong, J. H. Shih, S. D. Lin, T. C. Lu, T. R. Lin, *Sci. Rep.* **2017**, *7*, 39813.

[70] Y. H. Chou, B. T. Chou, C. K. Chiang, Y. Y. Lai, C. T. Yang, H. Li, T. R. Lin, C. C. Lin, H. C. Kuo, S. C. Wang, T. C. Lu, *ACS Nano* **2015**, *9*, 3978.

[71] Y. H. Chou, K. B. Hong, C. T. Chang, T. C. Chang, Z. T. Huang, P. J. Cheng, J. H. Yang, M. H. Lin, T. R. Lin, K. P. Chen, S. Gwo, T. C. Lu, *Nano Lett.* **2018**, *18*, 747.

[72] C. W. Cheng, Y. J. Liao, C. Y. Liu, B. H. Wu, S. S. Raja, C. Y. Wang, X. Li, C. K. Shih, L. J. Chen, S. Gwo, *ACS Photonics* **2018**, *5*, 2624.

[73] Y. H. Chou, Y. M. Wu, K. B. Hong, B. T. Chou, J. H. Shih, Y. C. Chung, P. Y. Chen, T. R. Lin, C. C. Lin, S. D. Lin, T. C. Lu, *Nano Lett.* **2016**, *16*, 3179.

[74] B. T. Chou, Y. H. Chou, C. K. Chiang, Y. M. Wu, T. R. Lin, S. D. Lin, T. C. Lu, *IEEE. J. Sel. Top. Quantum Electron.* **2015**, *21*, 1503106.

[75] Y. J. Liao, C. W. Cheng, B. H. Wu, C. Y. Wang, C. Y. Chen, S. Gwo, L. J. Chen, *RSC Adv.* **2019**, *9*, 13600.

[76] S. Sergent, K. Takiguchi, T. Tsuchizawa, H. Taniyama, M. Notomi, *ACS Photonics* **2020**, *7*, 1104.

[77] L. K. Van Vugt, S. Rühle, D. Vanmaekelbergh, D. *Nano Lett.* **2006**, *6*, 2707.

[78] J. Li, Y. Lin, J. Lu, C. Xu, Y. Wang, Z. Shi, J. Dai, *ACS Nano* **2015**, *9*, 6794.

[79] B. T. Chou, Y. H. Chou, Y. M. Wu, Y. C. Chung, W. J. Hsueh, S. W. Lin, T. C. Lu, T. R. Lin, S. D. Lin, *Sci. Rep.* **2016**, *6*, 19887.

[80] H. Y. Yang, S. F. Yu, G. P. Li, T. Wu, *Opt. Express* **2010**, *18*, 13647.

[81] C. Czekalla, C. Sturm, R. Schmidt-Grund, B. Cao, M. Lorenz, M. Grundmann, *Appl. Phys. Lett.* **2008**, *92*, 241102.

[82] P. J. Cheng, Z. T. Huang, J. H. Li, B. T. Chou, Y. H. Chou, W. C. Lo, K. P. Chen, T. C. Lu, T. R. Lin, *ACS Photonics.* **2018**, *5*, 2638.

[83] J. C. Johnson, H. Yan, R. D. Schaller, L. H. Haber, R. J. Saykally, P. Yang, *J. Phys. Chem. B* **2001**, *105*, 11387.

[84] C. Miao, H. Xu, M. Jiang, J. Ji, C. Kan, *Cryst. Eng. Comm.* **2022**, *22*, 5578.

[85] J. Li, M. Jiang, C. Xu, Y. Wang, Y. Lin, J. Lu, Z., Shi, *Sci. Rep.* **2015**, *5*, 9263.

[86] C. Miao, H. Xu, M. Jiang, Y. Liu, P. Wan, C. Kan, *Opt. Express* **2020**, *28*, 20920.

[87] K. Ma, X. Zhou, C. Kan, J. Xu, M. Jiang, *Phys. Chem. Chem. Phys.* **2021**, *23*, 6438.

[88] D. J. Gargas, M. E. Toimil-Molares, P. Yang, *J. Am. Chem. Soc.* **2009**, *131*, 2125.

[89] S. F. Yu, C. Yuen, S. P. Lau, W. I. Park, G. C. Yi, *Appl. Phys. Lett.* **2004**, *84*, 3241.

[90] P. Yang, H. Yan, S. Mao, R. Russo, J. Johnson, R. Saykally, N. Morris, J. Pham, R. He, H. Choi, *Adv. Funct. Mater.* **2002**, *12*, 323.

[91] M. H. Huang, S. Mao, H. Feick, H. Yan, Y. Wu, H. Kind, E. Weber, R. Russo, P. Yang, *Science*, **2001**, *292*, 1897.

[92] H. Beak, J. B. Park, J. W. Park, J. K. Hyun, H. Yoon, H. Oh, J. Yoon, *Appl. Phys. Lett.* **2016**, *108*, 263102.

[93] D. J. Gargas, M. C. Moore, A. Ni, S. W. Chang, Z. Zhang, S. L. Chuang, P. Yang, *ACS Nano* **2010**, *4*, 3270.

[94] Y. L. Ho, J. K. Clark, A. S. A. Kamal, J. J. Delaunay, *Nano Lett.* **2018**, *18*, 7769.

[95] Y. S. Zhao, A. Peng, H. Fu, Y. Ma, J. Yao, *Adv. Mater.* **2008**, *20*, 1661.

[96] F. Hjort, J. Enslin, M. Cobet, M. A. Bergmann, J. Gustavsson, T. Kolbe, A. Knauer, F. Nippert, I. Häusler, M. R. Wagner, T. Wernicke, M. Kneissl, A. Haglund, A. *ACS Photonics* **2021**, *8*, 135.

[97] Y. Zhang, H. Li, P. Li, A. Dehzangi, L. Wang, X. Yi, G. Wang, *IEEE Photonics J.* **2017**, *9*, 2400508.

[98] Y. He, Y. K. Song, A. V. Nurmikko, *Appl. Phys. Lett.* **2004**, *84*, 463.

[99] V. N. Jmerik, A. M. Mizerov, A. A. Sitnikova, P. S. Kop'ev, E. V. Ivanov, N. P. Tarasuk, N. V. Rzheutskii, G. P. Yablonskii, *Appl. Phys. Lett.* **2010**, *96*, 141112.

[100] R. Chen, H. D. Sun, T. Wang, K. N. Hui, H. W. Choi, *Appl. Phys. Lett*. **2010**, *96*, 241101.

[101] H. Y. Yang, S. F. Yu, J. Yan, L. D. Zhang, *Nanoscale Res. Lett.* **2010**, *5*, 809.

[102] J. X. Ding, J.A. Zapien. W. W. Chen, Y. Lifshitz, S. T. Lee, *Appl. Phys. Lett.* **2004**, *85* 2361. .

[103] H. Gao, A. Fu, S. C. Andrew, P. Yang, *Proc. Natl. Acad. Sci.* **2013**, *110*, 865.

[104] H. W. Choi, K. N. Hui, P. T. Lai, P. Chen, X. H. Zhang, S. Tripathy, J. H. Teng, S. J. Chua, *Appl. Phys. Lett.* **2006**, *89*, 211101.

[105] F. Qin, Q. Zhu, Y. Zhang, R. Wang, X. Wang, M. Zhou, Y. Yang, *Opt. Mater.* **2021**, *122*, 111663.

[106] M. H. Lo, Y. J. Cheng, M. C. Liu, H. C., Kuo, S. C. Wang, *Opt. Express* **2011**, *19*, 17960.

[107] C. L. Yu, Y. H. Hsiao, C. Y. Chang, P. J. Cheng, H. T. Lin, M. S. Lai, H. C. Kuo, S. W. Chang, M. H. Shih, *Sci. Rep*. **2020**, *10*, 7880.

[108] C. C. Chen, C. H. Chiu, S. P. Chang, M. Y. Kuo, J. K. Huang, H. C. Kuo, S. P. Chen, L. L. Lee, M. S. Jeng, *Appl. Phys. Lett*. **2013**, *102*, 011134.

[109] Y. C. Hsu, K. P. Sou, S. P. Chang, K. S. Hsu, M. H. Shih, H. C. Kuo, Y. J. Cheng, C. Y. Chang, *Appl. Phys. Lett.* **2013**, *103*, 191102.

[110] H. J. Choi, J. C. Johnson, R. He, S. K. Lee, F. Kim, P. Pauzauskie, J. Goldberger, R. J. Saykally, P. Yang, *J. Phys. Chem. B* **2003**, *107*, 8721.

[111] S. Sergent, B. Damilano, S.Vézian, S. Chenot, T. Tsuchizawa, M. Notomi, *Appl. Phys. Lett.* **2020**, *116*, 223101.

[112] Q. Zhang, G. Li, X. Liu, F. Qian, Y. Li, T. C. Sum, C. M. Lieber, Q. Xiong, *Nat. Commun.* **2014**, *5*, 4953.

[113] S. Sergent, B. Damilnao, S. Vézian, S. Chenot, M. Takiguchi, T. Tsuchizawa, H. Taniyama, M. Notomi, *ACS Photonics* **2019**, *6*, 3321.

[114] B. Damilano, P. M. Coulon, S. Vézian, V. Brändli, J. Y. Duboz, J. Massies, P. A. Shields, *Appl. Phys. Express* **2019**, *12*, 045007.

[115] W. C. Liao, Y. M. Liao, C. T. Su, P. Perumal, S. Y. Lin, W. J. Lin, C. H. Chang, H. I. Lin, G. Haider, C. Y. Chang, S. W. Chang, C. Y. Tsai, T. C. Lu, T. Y. Lin, Y. F. Chen, *ACS Appl. Nano Mater.* **2017**, *1*, 152.

[116] H. G. Park, F. Qian, C. J. Barrelet, M. Li, *Appl. Phys. Lett.* **2007**, *91*, 251115.

[117] G. Zhu, S. He, J. Li, J. Yuan, F. Qin, J. Li, X. Li, Y. Wang, *Opt. Commun.* **2020**, *474*, 126054.

[118] H. Xu, J. B. Wright, T. S. Luk, J. J. Figiel, K. Cross, L. F. Lester, G. Balakrishnan, G. T. Wang, I. Brener, Q. Li, *Appl. Phys. Lett.* **2012**, *101*, 113106.

[119] T. Kouno, K. Kishino, T. Suzuki, M. Sakai, *IEEE Photonics J.* **2010**, *2*, 1027.

[120] K. H. Li, Z. Ma, H. W. Choi, *Opt. Lett.* **2012**, *37*, 374.

[121] H. Liu, H. Zhang, L. Dong, Y. Zhang, C. Pan, *Nanotechnology* **2016**, *27*, 355201.

[122] L. C. Wang,Y. Y. Zhang, R. Chen, Z. Q. Liu, J. Ma, Z. Li, X. Y. Yi, H. J. Li, J. X. Wang, G. H. Wang, W. H. Zhu, J. M. Li, *Opt. Lett*. **2017**, *42*, 2976.

[123] H. Beak, C. H. Lee, K. Chung, G. C. Yi, *Nano Lett.* **2013**, *13*, 2782.

[124] H. Xu, J. B. Wright, A. Hurtado, Q. Li, T. S. Luk, J. J. Figiel, K. Cross, G. Balakrishnan, L. K. Lester, I. Brener, G. T. Wang, *Appl. Phys. Lett.* **2012**, *101*, 221114.

[125] Q. Li, J. B. Wright, W. W. Chow, T. S. Luk, I. Brener, L. F. Lester, T. Wang, *Opt. Express* **2012**, *20*, 17873.

[126] G. Zhu, J. Li, J. Guo, J. Dai, C. Xu, Y. Wang, *Opt. Express* **2018**, *43*, 647.

[127] Y. Ren, H. Zhu, Y. Wu, G. Lou, Y. Liang, S. Li, X. Gui, Z. Qiu, Z. Tang, *ACS Photonics* **2018**, *5*, 2503.

[128] J. B. Wright, S. Campione, S. Liu, J. A. Martinez, H. Xu, T. S. Luk, Q. Li, G. T. Wang, B. S. Swartzentruber, L. F. Lester, I. Brener, *Appl. Phys. Lett*. **2014**, *104*, 041107.

[129] S. Liu, B. Sheng, X. Wang, D. Dong, P. Wang, Z. Chen, T. Wang, X. Rong, D. Li, L. Yang, S. Liu, M. Li, J. Zhang, W. Ge, K. Shi, Y. Tong, B. Shen, *Appl. Phys. Lett*. **2018**, *112*, 231904.

[130] C. Y. Huang, J. J. Lin, T. C. Chang, C. Y. Liu, T. Y. Tai, K. B. Hong, T. C. Lu, H. C Kuo, *Nano Lett.* **2017**, *17*, 6228.

[131] S. S. Yan, A. Q. Chen, Y. Y. Wu, H. Zhu, X. H. Wang, C. C. Ling, S. C. Su, *RCS Adv.* **2017**, *7*, 21541.

[132] J. Heo, W. Guo, P. Bhattacharya, *Appl. Phys. Lett.* **2011**, *98*, 021110.

[133] S. Gradečak, F. Qian, Y. Li, H. G. Park, C. M. Lieber, *Appl. Phys. Lett.* **2005**, *87*, 173111.

[134] J. C. Johnson, H. J. Choi, K. P. Knutsen, R. D. Schaller, P. Yang, R. J. Saykally, *Nat. Mater.* **2002**, *1*, 106.

[135] Y. G. Wang, C. C. Chen, C. H. Chiu, M. Y. Kuo,. M. H. Shih, H. C. Kuo, *Appl. Phys. Lett.* **2011**, *98*, 131110.

[136] C. C. Chen, M. H. Shih, Y. C. Yang, H. C. Kuo, *Appl. Phys. Lett.* **2010**, *96*, 151115.

[137] W. C. Liao, S. W. Liao, K. J. Chen, Y. H. Hsiao, S. W. Chang, H. C. Kuo, M. H. Shih, *Sci. Rep*. **2016**, *6*, 26578.

[138] Y. Wang, L. Jiang, J. Chen, F. Liu, Y. Lai, *Mater. Lett.* **2017**, *193*, 228.

[139] K. Kirubanithy, S. K. Jayaraj, R. Beura, T. Paramasivam, *Environ. Nanotechnol. Monit. Manag.* **2022**, *17*, 100629.
